# Goal-Based Private Sustainability Governance and Its Paradoxes in the Indonesian Palm Oil Sector

**DOI:** 10.1007/s10551-023-05377-1

**Published:** 2023-03-13

**Authors:** Janina Grabs, Rachael D. Garrett

**Affiliations:** 1 Environmental Policy Lab, Department of Humanities, Social and Political Sciences, ETH Zürich, Sonneggstrasse 33, CH-8092 Zurich, Switzerland; 2 Universitat Ramon Llull, ESADE Business School, Av. Torre Blanca 59, 08172 Sant Cugat, Spain; 3 Department of Geography, Cambridge University, Cambridge, UK

**Keywords:** Private sustainability governance, Governance through goals, Paradoxical tensions

## Abstract

In response to stakeholder pressure, companies increasingly make ambitious forward-looking sustainability commitments. They then draw on corporate policies with varying degrees of alignment to disseminate and enforce corresponding behavioral rules among their suppliers and business partners. This goal-based turn in private sustainability governance has important implications for its likely environmental and social outcomes. Drawing on paradox theory, this article uses a case study of zero-deforestation commitments in the Indonesian palm oil sector to argue that goal-based private sustainability governance’s characteristics set the stage for two types of paradoxes to emerge: performing paradoxes between environmental, social, and economic sustainability goals, and organizing paradoxes between cooperation and competition approaches. Companies’ responses to these paradoxes, in turn, can explain the lack of full goal attainment and differential rates of progress between actors. These results draw our attention to the complexities hidden behind governance through goal setting in the corporate space, and raise important questions about the viability of similar strategies such as science-based targets and net-zero goals.

## Introduction

You cannot have both [no deforestation and smallholder inclusion], you can have one, you can have the other. And if you want to have both, you have to put some skin in the game and say, I will support change, and it will cost me. The problem is, if your neighbor doesn’t do it, your marketing team is going to say ‘why do we do that? We’re going to get hit and we’re going to lose market shares.’ It’s an uncomfortable balance to find. (LISC-04).

A growing number of companies are setting ambitious environmental and social sustainability targets. But what does this mean for impacts on the ground? Through a case study of zero-deforestation commitment implementation in the palm oil sector, this article argues that companies are likely to experience paradoxical tensions like the ones described in the opening quote when implementing private sustainability governance through goal setting. Depending on how they respond to these paradoxes, companies may set themselves apart as leaders or laggards in the sustainability space. But those who ignore paradoxical demands do so at their own peril, as contradictions will keep haunting them.

Goal setting is becoming an increasingly common form of transnational governance. Whereas the 1980s and 1990s were marked by international agreements specifying clear state obligations such as the Montreal or Kyoto Protocols, the turn of the millennium ushered in a focus on governance through goals in an effort to bring more actors on board for solving global environmental and social problems (Kanie & Biermann, 2017). Starting with the Millennium Development Goals, this trend became even more pronounced with the simultaneous announcement in 2015 of the Paris Climate Agreement (and its reliance on states’ Nationally Determined Contributions) and the United Nation’s Sustainable Development Goals (Biermann et al., 2017; McDermott et al., 2022; Rajamani, 2016).

In parallel to the development of governance through goals in the multilateral arena, private sector actors too have increasingly expressed their Corporate Social Responsibility and contributions to sustainable development and ecosystem conservation through goal setting as a sustainability strategy. In this strategy, companies make ambitious forward-looking commitments that cover their own operations and supply chains. They then rely on corporate policies and supplier codes of conduct—with varying degrees of cross-company and cross-sectoral alignment—to fulfill those promises. In this way, they create, disseminate, and enforce their own rules among other actors in their supply chains and business networks, making this strategy a form of *goal-based private sustainability governance* (Bjørn et al., 2021; Garrett et al., 2019; Grabs, 2022; Grabs et al., 2021; Thorlakson, 2018).

These goal-based approaches stand in contrast to historical private governance efforts focused on third-party certification schemes (e.g., Fairtrade or Rainforest Alliance), multi-stakeholder roundtables and standards (e.g., Forest Stewardship Council or Roundtable on Sustainable Palm Oil), and industry-wide codes of conduct (e.g., the chemical industry’s Responsible Care initiative) (Angel et al., 2007), which we categorize as “rule-based” private governance approaches. In such schemes the behavioral rules are co-created collectively (often through elaborate multi-stakeholder consultation processes) at the inception of the scheme, revised through formal procedures, and externally verified. Private actors then choose voluntarily to be bound by such rules by becoming an initiative member or becoming certified (DeFries et al., 2017; Meemken, 2020; Oya et al., 2018).

While governing through goals has been an increasingly important focus of global governance research (Biermann et al., 2017; Kanie & Biermann, 2017), it has received less attention in the analysis of interactions among private actors, or between private actors and civil society or state actors, i.e., “private governance” (Falkner, 2003, p. 72). This research gap is problematic because we may fail to foresee the unique ethical challenges that private governance efforts are likely to encounter when moving from rule-based to goal-based modes of operation. Such challenges may include inclusion and equity considerations (Grabs et al., 2021; McDermott et al., 2022), issues of goal tensions and trade-offs (Bernstein, 2017), and doubts regarding the effectiveness and ultimate goal attainment of such strategies (Young, 2017).

To better understand the opportunities and limitations of private governance through goals, we draw on paradox theory—a business ethics theory that focuses on “persistent contradiction[s] between interdependent elements” (Schad et al., 2016, p. 6) and competing demands on businesses—and ask: *What kinds of paradoxical tensions emerge when implementing goal-based private sustainability governance, and how do companies deal with such paradoxes?* We explore this question through an abductive study of the implementation of zero-deforestation commitments (ZDCs) in the palm oil sector of Indonesia, analyzing over 60 semi-structured expert interviews as well as meeting notes and primary documents. The palm oil sector has historically been a leading deforestation driver globally, especially in the biodiversity hotspot of Indonesia (Pendrill et al., 2019); simultaneously it is a key sector for the country’s economic development (Cramb & McCarthy, 2016; Padfield et al., 2016). This context sets the stage for multiple paradoxes to emerge.

We uncover both performing paradoxes (related to contradictory demands and goals) and organizing paradoxes (related to ways in which companies act; here specifically related to cooperation versus competition). While many large companies acknowledged and constructively managed their paradoxes, companies with less reputational exposure and capabilities tended to use defensive or destabilizing responses to justify their own inaction on commitment implementation. These paradoxes, ultimately, prevented companies from achieving 100% deforestation-free value chains. Still, companies continued to take action that changed the economic calculus of large commodity growers on the ground, which is seen to have slowed their rate of expansion into forested areas. Similar or related tensions are likely to characterize other types of goal-based private sustainability governance such as net-zero emission targets or the elimination of modern slavery. Private governance via goal setting may be particularly well suited for kick-starting innovation and sectoral transformations; yet requires sensitivity to goal conflicts and negotiations between stakeholders to converge on best practices that avoid unintended consequences.

By using a paradox perspective to analyze the goal-based private governance turn in the context of supply-chain policy implementation, our work opens up new research avenues of relevance to scholars of transnational governance and business ethics, and links the two fields closer together. It responds to the call to establish more links between paradox and sustainability research “by linking firms’ actions to systemic problems,” thereby allowing researchers to “complicate our understandings of nested and interwoven tensions” (Schad et al., 2019, p. 114). Our findings on core paradoxes that companies face in implementing ZDCs also have strong public policy implications, specifically for legislative proposals underway in the United States and European Union to enhance mandatory corporate due diligence and deforestation-free imports (Bager et al., 2021; Korte, 2021). Complying with such legislation will likely rely on similar implementation mechanisms and encounter similar challenges.

The section “Literature Review: Using a Paradox Perspective to Understand Tensions and Goal Conflicts in Private Governance Through Goal Setting” provides an overview of the private governance and paradox theory literatures and develops the research gap. Section “The Case Study: Zero-Deforestation Commitments in the Palm Oil Supply Chain” introduces the case study, Sect. “Methods and Data” describes our methods and data, and Sect. “Findings” summarizes the results. Section “Discussion” discusses the findings and Sect. “Conclusions” concludes the paper.

## Literature Review: Using a Paradox Perspective to Understand Tensions and Goal Conflicts in Private Governance Through Goal Setting

### The Goal-Based Turn in Private Sustainability Governance and Its Challenges

Private sustainability governance—that is, “interactions among private actors, or between private actors on the one hand and civil society and state actors on the other, giving rise to institutional arrangements that structure and direct actors’ behavior in an issue specific area” (Falkner, 2003, p. 72)—has risen in importance since the 1992 Earth Summit in Rio de Janeiro (Vandenbergh, 2013). In its first iteration, such governance occurred mainly through rule-based modes of operation such as third-party certification schemes, multi-stakeholder roundtables, and industry-wide codes of conduct (Angel et al., 2007; Fransen & Kolk, 2007). Research about such initiatives has focused, inter alia, on firms’ motivations to join (Bullock & van der Ven, 2020; Prakash & Potoski, 2006; Zeyen et al., 2016), the deliberative quality and legitimacy of the rule-making process (Arenas et al., 2020; Baumann-Pauly et al., 2017; Bowen, 2019; Cashore, 2002; Marin-Burgos et al., 2015; Schouten & Glasbergen, 2011; Schouten et al., 2012), the compliance of certified entities on the ground (Egels-Zandén, 2014; Malets, 2015; Wijen, 2014), and the likely effectiveness and additionality of participation (Aragòn-Correa et al., 2020; Dietz & Grabs, 2021; Dietz et al., 2019; Garrett et al., 2016; Oya et al., 2018). While it is challenging to briefly summarize such a vast and interdisciplinary literature, current research on private sustainability governance is coalescing around the conclusions that rule-based governance through certification schemes tends to lack context sensitivity (Bitzer & Schouten, 2022; Garrett et al., 2021; Oya et al., 2018), may create substantial costs that are both overt and hidden (LeBaron & Lister, 2021; Meemken, 2020; Oya et al., 2018), allows for the reproduction of unequal power relations (Grabs et al., 2020; Ponte, 2019), and is unlikely to shift producers’ behaviors sufficiently to bring about substantial environmental or social improvements (Aragòn-Correa et al., 2020; Carlson et al., 2018; DeFries et al., 2017; Dietz et al., 2022; Garrett et al., 2021; Meemken, 2020; van der Ven et al., 2018). In consequence, practitioners and researchers have advanced a call to go ‘beyond certification’ and explore new forms of private governance (Poynton, 2015; Thorlakson, 2018). Goal-based governance can be seen as an answer to this call, especially in light of broader civil society pressures on companies to make bold commitments (Garrett et al., 2019).

Like with other forms of private governance, the aim of goal-based private sustainability governance is to make a sector or industry more sustainable (Lambin et al., 2018). In that sense, it goes beyond single-company Corporate Social Responsibility or sustainable supply chain management where goals are company-specific and relatively unrelated to each other. While goals are set collectively, their definition tends to originate in negotiations between leading industry actors and civil society groups, with other industry actors forced to follow in order to avoid reputational damage (Bager & Lambin, 2022; Padfield et al., 2016). To garner attention and buy-in, goals are likely to be formulated in simple and absolute terms, such as ‘zero-deforestation’ or ‘zero-net emissions’. The key difference to rule-based private sustainability governance is that the pathway toward goal achievement tends to be flexible, with companies’ legitimacy and performance being evaluated on to what extent they achieve their goal, rather than whether they adhere to collectively set rules (Bjørn et al., 2021; McDermott et al., 2022). Still, companies create new internal procedures and pass demands to their suppliers and business partners, in the process creating “institutional arrangements that structure and direct actors’ behavior in an issue specific area” (Falkner, 2003, p. 72)—i.e., private governance. A growing literature has focused on the adoption, coverage, and design of such commitments (Chrun et al., 2016; Garrett et al., 2019; Gollnow et al., 2022; Heilmayr et al., 2020; zu Ermgassen et al., 2020), with few qualitative studies assessing their implementation (Cammelli et al., 2022; Lyons-White & Knight, 2018).

The existing literature indicates that governance via goal setting is conceptually different from its rule-based precursors. Examining governing through goals at the interstate level via the example of the Sustainable Development Goals (SDGs), Biermann et al. (2017, p. 26) note that this form of global governance is “new and unique for a number of characteristics such as the inclusive goal-setting process, the non-binding nature of the goals, the reliance on weak institutional arrangements, and the extensive leeway that states enjoy.” Table 1 shows that many of these characteristics also hold for goal-based private sustainability governance and set it apart from rule-based private governance.

**Table 1:** Characteristics of goal-based private sustainability governance as compared to global governance through goals and rule-based private sustainability governance

	Global governance through goals	Goal-based private sustainability governance	Rule-based private sustainability governance
Goal-setting process	Inclusive goal-setting process	Goal-setting process is collective, but not inclusive, as it is dominated by negotiations between leading NGOs and corporate actors	No one overarching goal; components of ‘sustainability’ co-decided through standard design process
Binding or non-binding	Non-binding nature of the goals	Perceived binding-ness of goals depends on reputational risk and stakeholder evaluation	Rules are binding for participants; but possibility to leave scheme
Amount of leeway for implementation	States enjoy extensive leeway in implementing goals	Companies enjoy extensive leeway in implementing goals, though subject to stakeholder evaluation and progressive alignment	Companies have moderate leeway only if standard allows for it; otherwise dependent on auditor interpretation
Institutional arrangements	Reliance on weak institutional arrangements	Reliance on weak institutional arrangements	Building of relatively strong institutional arrangements via roundtables and standard organizations
References	Biermann et al. (2017), Kanie & Biermann (2017), Vijge et al. (2020)	Bager & Lambin (2022), Bjørn et al. (2021), Garrett et al. (2019), Grabs et al. (2021), Lyons-White et al. (2020), McDermott et al. (2022)	Aragòn-Correa et al. (2020), Arenas et al. (2020), Auld (2010), Baumann-Pauly et al. (2017), Cashore et al. (2004), Grabs et al. (2020), Tröster & Hiete (2018)

The different characteristics of goal-based compared to rule-based governance lead to important differences in implementation processes and outcomes. On the positive side, Young (2017) argues that in comparison to rules that create indefinite behavioral prescriptions, specific, time-bound targets may galvanize more enthusiastic action by a greater number of actors which join forces to each contribute to the goal in their own way. Yet, the success of governing through goals relies on “the increasing formalization of commitments, the establishment of clear benchmarks, and the issuance of formal, measurable pledges” that may “cause embarrassment or loss of face in case of non-compliance” (Biermann & Kanie, 2017, p. 300).

Other authors caution that goals may contain normative ambiguity (Vijge et al., 2020), that there may be tensions and tradeoffs between various goals (Bernstein, 2017), and that a prioritization of goals may be needed (Spangenberg, 2017). McDermott et al. (2022) highlight that target setting without concern for local contexts may confuse the means and ends of transformation and reinforce unequal power dynamics. In response, they launch a call to foreground equity considerations in both research and practice. Grabs et al. (2021) echo this call by pointing in particular to potential effectiveness-equity tensions at the heart of zero-deforestation commitment implementation. This article responds to these concerns and hones in on goal tensions and reactions to them by corporate actors by using a theoretical lens new to transnational governance research: the paradox perspective.

### The Paradox Perspective

Originating in business ethics and organization studies scholarship, the paradox perspective is a notable departure from assertions that corporate sustainability can be achieved through the business case logic or win–win strategies such as the creation of shared value (Carroll & Shabana, 2010; Kleine & von Hauff, 2009; Porter & Kramer, 2011). Instead, organizational tension or paradox theory (we use the shorthand ‘paradox perspective’ to refer to both streams of literature) proposes that it is valuable to acknowledge contradictory demands and shine greater light on creative ways in which organizations can attend to them (Lewis, 2000; Scherer et al., 2013; Smith & Lewis, 2011).

Conceptually, scholars distinguish between the broad category of tensions, “the clash of ideas or principles or actions and […] the discomfort that may arise as a result,” and the subcategory of paradoxes, which are “pragmatic or interaction-based situations in which, in the pursuit of one goal, the pursuit of another competing goal enters the situation (often without intention) so as to undermine the first pursuit” (Stohl & Cheney, 2001, pp. 353–354). Hence, according to current usage in the literature, not all tensions are paradoxical, but all paradoxes are tensions (Hahn et al., 2015; Stohl & Cheney, 2001). The interdependence between elements and longevity of tensions make paradoxes different from ‘either/or dilemmas’ and ‘trade-offs’ of “competing choices, each with advantages and disadvantages” (Smith & Lewis, 2011, p. 387), where businesses may prioritize one element over another (Putnam et al., 2016). Dilemmas can however become paradoxical “when options are contradictory and interrelated such that any choice between them is temporary and tension will resurface” (Smith & Lewis, 2011, p. 387). The literature differentiates between corporate tensions or paradoxes of belonging (tensions of identity), learning (managing diverse knowledge and innovation processes), organizing (implementing contradictory processes), and performing (pursuing competing goals and satisfying multiple stakeholders) (Smith & Lewis, 2011).

We aim to understand both what types of paradoxes emerge and what range of strategies different companies pursue when faced with the same tensions and paradoxes. According to the literature, companies may respond to tensions and paradoxes through constructive (also called strategic) or defensive responses (Pinkse et al., 2019), each of which includes a range of practices (Jarzabkowski & Lê, 2017). Constructive responses “accept tensions in corporate sustainability and pursue different sustainability aspects simultaneously even if they seem to contradict each other” (Hahn et al., 2014, 2015, p. 297; Van der Byl & Slawinski, 2015). Defensive responses refer to “defense mechanisms [through which firms aim to avoid sustainability tensions] that can cause good intentions to result in undesired outcomes” (Ferns et al., 2019; Iivonen, 2018; Schad et al., 2016, p. 39).

A small number of recent studies have used a paradox lens in sustainable supply chain management (Brix-Asala et al., 2018; Longoni et al., 2019; Xiao et al., 2019). Most of these describe perceived performing paradoxes between ‘sustainability’ (combining environmental and social issues) and business performance/economic goals such as cost competitiveness (Zhang et al., 2021), and examine single company case studies. Xiao et al. (2019) found that the buying firm they studied moderated paradoxical tensions by either suppressing sustainability goals or ‘contextualizing’ sustainability by developing responses appropriate for emerging market contexts. Brix-Asala et al. (2018, p. 424) found that tensions in their case study were mainly addressed “via pro-active and direct supplier and stakeholder engagement.”

While these contributions provide valuable insights into corporate strategies to navigate paradoxes related to sustainable supply chain management, the reliance on single company case studies has several limitations. When focusing primarily on company-internal dynamics, paradoxes and responses resulting from companies’ interaction with competitors and other sectoral actors might be overlooked. Furthermore, it is unclear how generalizable insights stemming from single company cases are, and to what extent other companies might act in similar or different ways. By using a sectoral scope that is sensitive to companies’ interactions and interviewing a wide range of stakeholders, our study addresses these limitations.

## The Case Study: Zero-Deforestation Commitments in the Palm Oil Supply Chain

**Fig. 1:**
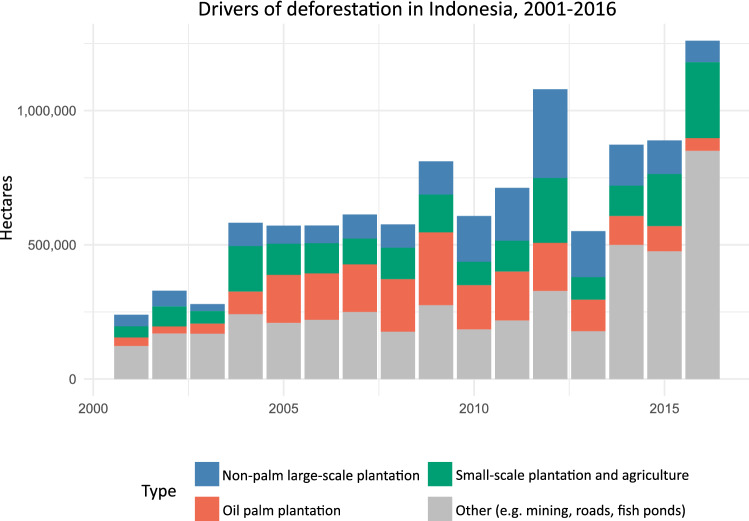
Drivers of deforestation in Indonesia, 2001–2016. Large-scale oil palm plantations drove 23% of deforestation during this time period, with peaks in 2008–2009, when they accounted for around 40% of national deforestation. Data from Austin et al. (2019), own illustration

The rapid expansion of palm oil across South-East Asia is associated with the loss of primary forests, the habitats of endangered animals, and carbon emissions from converted peat land (Gaveau et al., 2019). Between 1995 and 2015, Indonesia lost an estimated 117,000 ha of forest annually due to oil palm expansion (Austin et al., 2017), accounting for around one-quarter of all deforestation in the country (Austin et al., 2019) (Fig. 1).

In the absence of deforestation restrictions, models projected the conversion of a further 7.5–21.1 million ha of Indonesian forests for oil palm between 2010 and 2030 (Mosnier et al., 2017). In response, from 2010 onward civil society organizations made a concerted push for corporate action to eliminate deforestation, using collective arenas such as the Consumer Goods Forum or the Tropical Forest Alliance (Lister & Dauvergne, 2014). To date, almost 300 companies have made palm oil-specific sourcing commitments (often framed as NDPE [No Deforestation, No Peat, No Exploitation] commitments), and companies with ZDCs refine around 83% of the palm oil produced in Southeast Asia (ten Kate et al., 2020). ZDCs thus have the potential to become powerful private governance tools through the strategic use of market power if implemented effectively along the complex supply chain (Larsen et al., 2018).

**Fig. 2:**
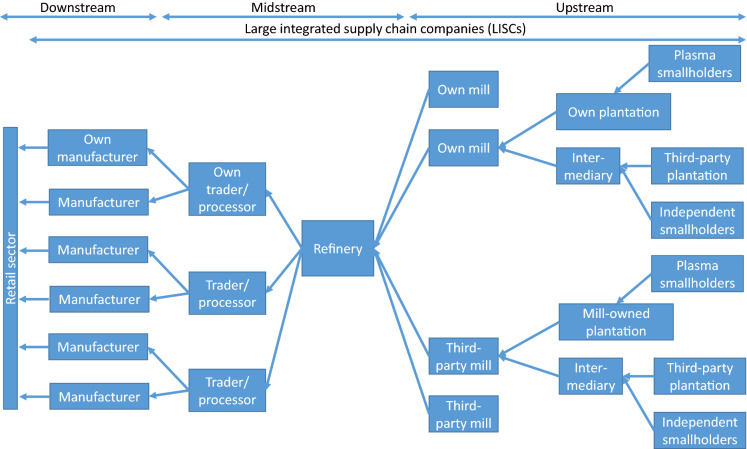
This schematized palm oil supply chain from the view of a large integrated supply chain company’s refinery illustrates the high complexity and multiple levels of actors in the sector, who need to work collaboratively to disseminate and enforce corporate supplier policies. Own illustration, adapted from Lyons-White and Knight (2018)

The palm oil supply chain is made up by a mixture of large integrated supply chain companies (LISCs) and independent actors (Lyons-White & Knight, 2018) (see Fig. 2). Most refining and processing companies source from both their own palm oil mills and plantations as well as from third-party mills. Mills in turn source their fresh fruit bunches (FFB, the primary agricultural good) from their own plantations, associated (‘plasma’) smallholders, as well as independent (smallholder) farmers, who are linked to mills by informal intermediaries (Cramb & McCarthy, 2016). Downstream, the palm oil supply chain is equally complex. The refined oil and derivatives are used in a wide variety of goods, including direct consumption as cooking oil, as ingredient for food products, in derivative form for cosmetics and cleaning products, as well as major biodiesel component (Lyons-White & Knight, 2018).

**Fig. 3:**
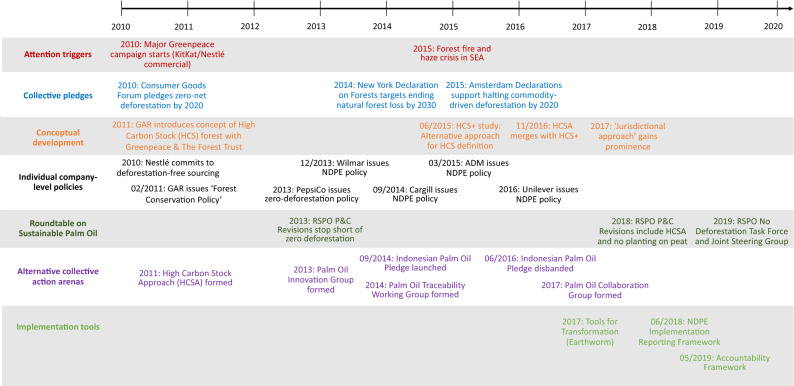
Timeline of development of ZDC implementation concepts, collective action arenas, and tools in the palm oil sector. Own illustration

In response to this complexity, a diverse set of implementation mechanisms has emerged. Figure 3 provides an overview of the development of ZDC concepts and tools in the palm sector in the last decade. It shows that most ‘No Deforestation, No Peat, No Exploitation’ (NDPE) commitments^1^ and sourcing policies were adopted between 2013 and 2016 (Larsen et al., 2018). These zero-deforestation commitments exhibit many of the characteristics of goal-based private sustainability governance. First, goals are adopted on a company-by-company basis, often in response to external pressures. In this case NGOs targeted individual companies and pushed for them to adopt zero-deforestation commitments and associated policies. Second, as the zero-deforestation goal was a new and hitherto untried concept, companies had a lot of leeway to implement their commitments, yet little existing evidence to guide their efforts and little input from producing country actors. Third, stakeholders evaluated companies’ goal attainment performance selectively by focusing on the largest players in both traded volume and associated deforestation. Fourth, the main certification scheme for oil palm globally, the Roundtable on Sustainable Palm Oil (RSPO), only integrated zero-deforestation criteria in its 2018 revision. This meant that committed actors had to rely on alternative, informal institutional arrangements to coordinate and share best practices (Cheyns et al., 2020). As a result, there has been a proliferation of different NDPE policies with no formalized rule set. See Table 7 in the Appendix for interview quotes that illustrate these characteristics.

## Methods and Data

We use an abductive approach to case research known as systemic combining, whereby the “theoretical framework, empirical fieldwork, and case analysis evolve simultaneously” (Dubois & Gadde, 2002, p. 554) in an “emergent logic” (Piekkari et al., 2009, p. 572) through an iterative and recursive process of “double-fitting data and theories” (Timmermans & Tavory, 2012, p. 179). We follow Piekkari et al. (2009, p. 569) in defining a case study as “a research strategy that examines, through the use of a variety of data sources, a phenomenon in its naturalistic context, with the purpose of ‘confronting’ theory with the empirical world.” We conduct a single, deep probing case study of the implementation of private deforestation governance in the global palm oil production network, with a particular emphasis on actors connected to the Indonesian palm oil supply base. We see this as one particular example of goal-based private sustainability governance in global supply chains more broadly, which allows us to propose theoretical advancements that may inform other such cases.

In our data collection process, we started with a broad review of grey literature and corporate communications (via websites and CSR reports) on ZDC implementation in the palm oil sector. The first author took notes in 12 in-person and virtual workshops and webinars (summarized in Table 4 in the Appendix) which took place between 2019 and 2020. The first author also conducted 62 interviews (average 60 min, range 45–120 min of length) with companies along the supply chain, stakeholders, and supporting organizations and industry experts in the global and Indonesian palm oil sectors conducted between September 2019 and January 2021. 16 interviews were preliminary in scope to inform further directions of data collection, while 46 interviews were recorded, transcribed, and coded in NVivo to be analyzed for key themes and insights.

Fourteen of the coded interviews were conducted in person in Indonesia during a fieldwork trip that subsequently had to be cut short due to the COVID-19 pandemic. The remainder of the interviews were conducted virtually via Skype or Zoom, building on connections and contacts made in the field. As this change happened at the same time as a broader global move to virtual meetings due to work-from-home orders, all interviewees were comfortable with the video-conferencing software and it was possible to create rapport similar to in-person expert interview settings (cf. Gray et al., 2020; Howlett, 2021). We further added the notes from the workshops and webinars to the material to be coded, and drew on the grey literature and corporate communications when validating our coded materials.

We sampled organizations and individuals on the basis of a comprehensive stakeholder mapping of the ZDC governance and implementation space in the palm oil sector. After constructing this map (represented in the Appendix, Fig. 5), which represented all organizations involved in the main multi-stakeholder initiatives or collective efforts to reduce deforestation, and verifying that it included the major companies in the sector known to have zero-deforestation commitments, we reached out to representatives of all organizations via personal connections, snowball sampling, or online (e.g., LinkedIn, email if available).

We interviewed all companies that responded positively to our inquiry for an interview. This strategy necessarily implies that the perceptions of non-respondents are not captured in this paper, which may be a limitation to the results found and an impetus for future work. However, we addressed this limitation in two ways. First, we confirmed that our interviewee pool includes a variety of company sizes, supply chain positions, levels of forward/backward integration, and also includes companies notably less enthusiastic about ZDC implementation. Second, we supplemented our interviews with webinar notes as well as primary document analysis to capture alternative voices. All interviews were collected anonymously and will be referred to by the codes shown in Table 2 (see also Table 5 in the Appendix for more information about the interviews).

**Table 2:** Overview of coded interviews and the types of stakeholders interviewed

Number of interviewees	Code	Type
*Supply chain members*		
5	GRO	Grower
2	SHO	Smallholder organization
4 companies, 6 interviews	LISC	Large integrated supply chain company
4	TRA	Trader/processor
5	CGM	Consumer goods manufacturer
*Stakeholders*		
8	NGO	Environmental and/or social NGO
*Supporting organizations and industry experts*		
6 organizations, 8 interviews	TSO	Technical support organization
2	DEV	Development organization
1	MSO	Multi-stakeholder organization
5	CON	Consultant
46		Total

The semi-structured interviews with companies and supporting organizations used the focal question “On the basis of your organization’s perspective, please describe your impression of the current state of ZDC/NDPE implementation. I am particularly interested in the types of strategies that companies use to fulfill their commitments, as well as challenges and best practices to overcome such challenges on the ground, and future trends in this area,” and followed up via further questions to clarify strategies, challenges, and best practices mentioned by interviewees. Our interviews with NGOs and consultants were focused on further understanding the different demands on companies, and over the course of the interviews also served to probe their perception of commonly mentioned sustainability challenges raised by industry actors and supporting organizations in an effort to separate commonly acknowledged paradoxes from paradoxes that were used to justify inaction.

Over the course of the interviews, it became increasingly apparent that many actors in the zero-deforestation governance space perceive themselves—and others—as caught “between a rock and a hard place” (LISC-04) when it comes to meeting diverse demands and achieving sustainability. The recurrent mentions of “tensions,” “dilemmas,” and needs to find a “delicate” (NGO-01, NGO-02) or “uncomfortable balance” (LISC-04) when making things that “don’t really go hand in hand [still] go hand in hand somehow” (TRA-04) led us to probe whether paradox theory might be an appropriate frame to assess and explain ZDC implementation challenges. Table 8 documents the inquiry which confirmed that many informants’ experience of issues and approaches to solutions tended to be expressed more frequently via the use of words related to seemingly contradictory, but co-existing tensions (e.g., yet, but, however, balance, on one hand/on the other hand) than words related to either/or dilemmas (such as tradeoffs, choice, resolve) (following Smith, 2014). This language justified our use of paradox theory as a lens through which to understand why companies were unable to meet their zero-deforestation commitment targets.

Subsequently, we identified 17 key issues that arise in the implementation of ZDCs in the palm oil sector that represented tensions between various demands or logics based on three criteria: (a) salience (in the perception of the interviewee; we assessed this through a combination of the length of time spoken about the issue and the expressed importance of the challenge); (b) tension between conflicting demands; and (c) multiple informants mentioning it. We then identified which demands constituted the conflicting poles for each issue, and assessed to what extent the poles were strongly interrelated and mutually reinforcing. If this was strongly and clearly the case, we speak of a paradox; if poles were only weakly interrelated, we speak of a tension.

In a next step, we categorized them in the typology of tensions/paradoxes outlined by Smith and Lewis (2011) (tensions/paradoxes of belonging, learning, organizing, and performing). This allowed us to identify both performing and organizing tensions/paradoxes that occurred in this case of goal-based private governance (overview provided in Table 9). In contrast, we did not identify paradoxes of belonging or learning in this study. This may be due to our focus on organizational strategies versus the values and identities of individual employees (which may have elicited paradoxes of belonging), and our prioritization of current implementation strategies versus a long-term strategic outlook (which may have elicited paradoxes of learning). After going back to the literature to review how previous authors had conceptualized company responses to tensions and paradoxes, we reviewed our data and coded response types in relation to the relevant issue and company, which resulted in Table 10 and the overview in Sect. “Responses to Paradoxical Tensions.” Table 3 summarizes our abductive analytical process in detail.

**Table 3:** Abductive analytical process of moving between data and theory to arrive at research findings, following Smith (2014)

	Stage	Analytical activities	Output
Data	Develop thick description to generate initial insights	First round of in-vivo coding to structure insights on the case Creation of timeline of activities and tools	Thick case description Timeline of tools and activities (Fig. 3) Overview of challenges related to deforestation (Table 6) Identification of themes related to challenges, tensions and contradictions
Theory	Review literature that touches on tensions and contradictions	Literature search of theories that build on tensions between stakeholder demands Identification of paradox theory; review of major contributions as well as applications to sustainability and sustainable supply chain literature	Overview of definition and framing of tensions and paradoxes, importance to conversation in business ethics Identification of paradox lens as promising theoretical approach to explain findings
Data	Identify key issues, poles of tension, classification as paradoxes	Generate a list of key issues that represented tensions between various demands or logics based on three criteria: (a) salience; (b) tension between conflicting demands; (c) multiple informants mentioning it Code issues using in-vivo codes or short phrases Cluster and incorporate literature Return to raw data to confirm all instances of issues Identify language that informants use to describe their understanding of issues; confirm paradox frame Identify poles of tension for each key issue Return to raw data to confirm all instances of demands/poles of tension Spatial ordering of issues between common poles Confirmation of interrelatedness of poles in each key issue; categorization as tension or paradox Simplification of poles, types of tensions, and visualization using commonly used categories	2 themes describing informants’ experience of issues (Table 8) List of 17 key issues representing (paradoxical) tensions (Table 9) Overview of poles of performing and organizing tensions (Fig. 4) Identification of organizational responses to tensions as key determinant of differential success of ZDC implementation
Theory	Return to literature to better understand responses to tensions	Focused literature search on responses to tensions and paradoxes	Identification of major types of responses and their description
Data	Identify response types. Classify responses by issue, actor, observer	Return to raw data and identify instances where companies responded to tensions and paradoxes Classify responses according to major type, issue, actor, and who described the response	Overview of three types of responses (Sect. “Responses to Paradoxical Tensions”) Examples for each type of response for selection of issues (Table 10)
Theory	Review literature to better understand the type of private sustainability governance analyzed	Focused literature review and identification of the literature on governance through goals and its characterization	Identification of main characteristics of governance through goals Comparison between governance through goals, goal-based private sustainability governance, and rule-based private sustainability governance (Table 1)
Data	Understand link between goal-based private sustainability governance and paradoxes	Return to raw data to confirm applicability of goal-based private sustainability governance Assessment and theorization of propensity for goal-based governance to lead to paradoxes based on case study	Interview-based confirmation of goal-based private governance characteristics (Table 7) Proposed theoretical link between goal-based governance and paradox theories (Discussion)
Integration	Incorporate data and literature to create final analysis	Combine data on sustainability initiative implementation, identified tensions, and responses to describe overall challenges and approaches to goal-based private sustainability governance	Final analysis

## Findings

### Performing and Organizing Paradoxes in the Context of ZDC Implementation

Implementing palm oil ZDC policies involves four common steps: (1) Identifying one’s upstream suppliers via traceability efforts; (2) disseminating policies and expectations to suppliers via workshops and one-on-one engagement; (3) monitoring the land owned by suppliers for indications of forest cover loss, using satellite imagery and field verification; and (4) responding to potential cases of non-compliance via grievance mechanisms that serve to influence suppliers to change their practices or exclude them from the supply chain (CGM-03, GRO-03, LISC-04, NGO-03, TRA-01, TSO-02, TSO-04, TSO-06).

**Fig. 4:**
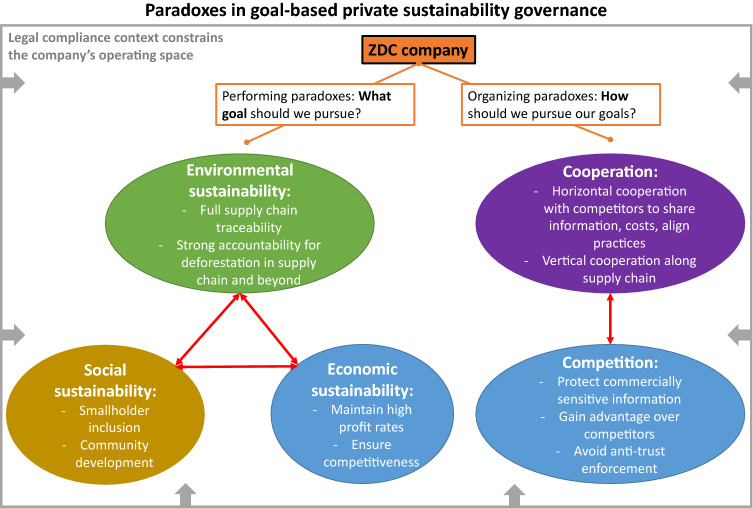
Companies face interrelated and conflicting demands coming from all sustainability dimensions and need to balance between two modes of operating while embedded in a challenging legal compliance context

Our interviews revealed that aiming to pursue those steps crucial for the singular goal of environmental sustainability comes into tension with the social sustainability goals of smallholder inclusion and community development as well as the goal of economic sustainability. These triple-bottom-line paradoxical tensions occurred within a challenging legal context in Indonesia in which legal compliance limited the operating space available to companies to find creative approaches to moderate tensions. In addition, companies found that they could reach certain goals—such as full supply chain traceability or exercising effective pressure to stop deforesting actors—only via close cooperation, both along the supply chain and between sectoral actors. Yet, this came into tension with their usual mode of competition, creating organizing paradoxes (see Fig. 4).

We found a total of 17 tensions between the above elements in the case study (compare Table 9). In the following, we hone in on three exemplary cases that illustrate these dynamics best: Smallholder inclusion in zero-deforestation supply chains (a performing paradox between the competing goals of environmental and social sustainability); the management of deforestation-related grievance cases (a performing paradox between the competing goals of environmental and economic sustainability); and the alignment of company action (an organizing paradox between modes of competition and cooperation).

#### Environmental-Social Paradoxes: The Case of Smallholder Inclusion

Smallholders^2^ manage around 50% of the global oil palm land (Byerlee et al., 2016), and an estimated 40% of oil palm area in Indonesia (Direktorat Jenderal Perkebunan, 2016). For many such smallholders, oil palm fruit sales constitute an important—or their only—source of livelihood (Jelsma et al., 2017). Ensuring that smallholders are included in sustainable palm oil supply chains and communities benefit from economic development is thus an important demand by social NGOs, and forms part of oil palm growers’ social license to operate (MSO-01, SHO-01, SHO-02, CON-05, NGO-08).

However, ensuring smallholder inclusion in deforestation-free value chains is seen as impossible to achieve simultaneously, at least in the short term (compare our opening quote). Yet, while these two poles are in conflict—as shown in more detail below –, they are also mutually interdependent. Many value chain actors assert that smallholders are currently driving deforestation, more so than established concessions (CON-01, LISC-03, NGO-02, TSO-07). Without smallholder inclusion, there can thus be no environmental sustainability. Similarly, without environmental sustainability (via deforestation reduction), the social sustainability and livelihoods of smallholders may be threatened in two ways: They may lose important market access, but they also depend on the forest for ecosystem services that regulate their microclimate, access to water and to alternative livelihood options. This is thus a classic example of paradoxical tensions.

In particular, downstream traceability expectations come into conflict with the stakeholder goal of smallholder inclusion (NGO-06). The increasing demand for fully traceable palm oil tends to be met via supply chain simplification, for instance via a so-called “1:1:1” strategy whereby all palm oil is only handled in one plantation, one mill, and one refinery. Such requests strongly favor the large integrated players (CGM-04, LISC-04). In contrast, mapping out a given mill’s smallholder supply base is challenging. Mills may purchase supply from thousands of ever-changing growers and there is no centralized database of smallholder land records (CGM-02, CON-05, GRO-03, TSO-03, TSO-05, LISC-04). Even if traceability is achieved in one time period, it is still not necessarily assured moving forward: “there will be hundreds if not thousands [of smallholders] for each mill, they will come and go, the database will never be accurate. […] Even if you have 10% of the smallholders who are in the wrong area supplying to you, that’s it, your credibility in terms of NDPE compliance goes away” (LISC-04). One trader raised the potential resulting supply chain exclusion of independent smallholders as a key concern: “we’re trying to resist and try to explain more that the outcome is not desirable. But unless we have a [GPS] coordinate for the smallholders, they’re saying, oh, it doesn’t meet our policy, and so you have to change or else we risk losing our whole business. So it’s usually difficult and damaging—at what point does one risk one’s own business being dropped at the expense of trying to defend the independent mills?” (TRA-03).

Beyond the difficulties of tracing smallholders, dealing with so-called ‘off-concession deforestation’ is seen as a key dilemma (TSO-03). For moral and political reasons, many zero deforestation companies officially do not use supply chain exclusion with respect to smallholders (see, for instance, Sime Darby (2019)). Instead, they prefer to map smallholders in their supply base and work with them to achieve better compliance in the future. However, the diversity of the so-called smallholders makes this approach a challenge too (GRO-02). Beyond the stereotypical family farmer with 1–2 ha of land, and beyond even the legal definition of Indonesian smallholders of up to 25 hectares, non-concessionaires in the palm oil business could hold hundreds, even thousands, of hectares of palm oil and nominally be considered ‘smallholders’ (CON-02, CON-05, LISC-04, LISC-05, NGO-01, TSO-02, TSO-07). Such *petani berdasi*, or absentee farmers, tend to be individuals from wealthy backgrounds, retired politicians, officials, or military officers, who may have accrued much of their area by consolidating smallholder plots (GRO-02, SHO-02, TSO-03). Individuals in their family or social network may also be nominal land owners, while the actual control over the land use strategy lies with the *petani berdasi*.

This poses a number of challenges to companies who want to engage with their so-called independent smallholder base. First, the individuals on the ground have little actual decision-making power (SHO-02); and second, the mapping and engagement efforts might unveil the legal non-compliance of politically powerful actors who prefer to remain in the shadows (SHO-02, TSO-03). Indeed, such mid-size actors might be most responsible for deforestation outside of concessions, more so than ‘small’ smallholders (LISC-05, LISC-04, NGO-06, SHO-02, TSO-03, TSO-06). Trying to change their practices, or even publicly drawing attention to them, might thus constitute a serious business risk and impede companies to operate in the region (CGM-04, LISC-05). Here, smallholder inclusion efforts may result in serious tensions with supply chain accountability.

#### Environmental-Economic Paradoxes: The Case of Grievance Resolution

We find a second paradoxical tension between environmental sustainability (particularly focused on accountability for deforestation events) and economic sustainability. The increased reputational and material risk of companies that do not enforce their own policies in their supply chain makes environmental sustainability a key factor for their economic sustainability. Simultaneously, only companies that can survive in the marketplace will be able to influence action on the ground in the long run, making economic sustainability a precondition for environmental sustainability. These mutually interdependent elements, however, are in conflict when it comes to the issue of how to respond to cases of deforestation by their suppliers, in particular whether and how quickly to suspend suppliers.

Most practitioners acknowledged that it was important to engage suppliers rather than immediately exclude them (CGM-01, CGM-05, LISC-01, LISC-06, NGO-03, TRA-02, TRA-03, TRA-04, TSO-04, TSO-06, TSO-07), given that there are continued opportunities to sell into the leakage market, and that buyers only had tenuous influence over the actions of third-party suppliers (GRO-03), which quickly waned once they exclude suppliers (CGM-01, LISC-04). Still, NGOs have been pushing for more uniform rules and decisiveness when dropping suppliers, especially given that “there’s still a lot of companies linked to deforestation within these supply chains” (NGO-08, NGO-06). On average, buyers only resolve between 30–50% of all grievance cases, while many cases stay open for years and see limited action (see also Table 11 in the Appendix).

This reluctance to drop suppliers may also be due to a motivation to maintain a broad supply base. In the Indonesian market in particular, since 2015 companies have operated in a sellers’ market as local refining capacity increased (LISC-05). Thus, “it’s a big thing to suspend one of your suppliers because that volume is essentially lost for the next couple of years. There is no volume on the markets, which you could easily take [up] to fill that void” (LISC-05). As maintaining processing facilities at full capacity is a key competitiveness criterion for midstream actors, a prerequisite for actually putting zero deforestation commitments into action requires the assurance “that they will still have enough supply for their mill even after their no deforestation commitment” (TSO-05). As one consultant observed, “[not dropping suppliers] is also about not destroying themselves, keeping their profitability, and effectively desiring a level playing field” (CON-04). A trader noted that “when we engage them, we want them to rectify, give them corrective measures so that they’re still in the supply chain because at the end of the day, we want as many mills to be compliant with the NDPE as possible, because we need them basically, we need the oil, that is our business. We are not NGOs” (TRA-02). Yet, this meant that “a number of the traders [are] not even suspending supply contracts when there [are] egregious violations” (NGO-06). In consequence, experts talked about a “two-tier NDPE market [where] you have the same policies, but some companies really implement them, and some not at all”—due to a combination of commercial reasons, personal relationships with growers, as well as political considerations (NGO-08).

#### Competition-Cooperation Paradoxes When Aligning Company Action

Finally, we can identify paradoxical tensions between competition (over both legitimacy and market leadership, compare Hahn and Pinkse (2014)) and cooperation to develop and implement NDPE policies. These poles, again, are mutually interdependent: a company can only credibly compete for market leadership if it uses procedures that others accept as legitimate (via cooperation and mutual alignment) and if it cooperates with others along the supply chain to share information and knowledge. This was recognized by the industry, as interviewees agreed that “over the past six years, we’ve seen unprecedented collaboration across the industry. Competitors, peers, suppliers, customers working together, that would have never happened beforehand” (TRA-01). Simultaneously, such cooperation is ultimately motivated by companies’ competitive tendencies. First, “most companies try to position themselves as being ahead of the curve. Being the better one of a not so great industry, and being then the reliable partner where the buyer can safely come to and get the volumes” (LISC-05). Others try to prevent being seen as laggards and “tend to be a fast follower. So we really look for indication from our peers who might be a little bit further along on their journey, to understand what they’re doing, because this is a very collaborative industry. […] Staying close to our peers and to industry groups is incredibly important” (CGM-05). At the same time, there exist persistent tensions between cooperation and competition.

We find a first example of this in grievance management. NGOs have encouraged companies to use the concerted market power of buyers more effectively, since “the successes that you see, it’s when there’s been collective action by all buyers” (NGO-08). Yet, such collective action—where companies simultaneously suspend a non-compliant supplier, thereby exerting maximum pressure—has been relatively rare, and only in cases with substantive media attention. This is partially because specific information on supplier contracts is still considered commercially sensitive information (TSO-08), but also because “there’s issues of anti-competition and anti-trust,” such that “buyers will often not want to announce that they have stopped buying from a grower because of the political ramifications” (NGO-08). Buyers further note that “you can’t have discussions [on how to deal with grievances] due to anti-competition law” (TRA-01, TRA-03), which makes alignment of responses difficult. The concern about following competition law was also voiced when companies considered sharing data (TRA-01, TSO-07) and aligning on operating procedures, as “there could be no collusion, in terms of setting a set of conditions” (TRA-03).

Still, as one trader mentioned, concerns about cooperating are driven by “competition among our competitors as well. You know, we want to get to more supply because at the end of the day, it is not just about sustainability, it is about business as well” (TRA-02). In particular, “if you have your traffic light chart where you kind of categorize suppliers into green, yellow and red, the competition around the greens [i.e., the sustainable supply base] is huge” (LISC-05). This means that companies are reluctant to share information on their engagement strategies and best-performing mills “because we want to engage with these mills so that we can hold these mills, without losing them, because of their importance to us, strategically, or probably for our competitors, the mills [are] important to them strategically” (TRA-02). On the other hand, when it comes to grievance management and in particular the definition of what is necessary for a supplier to re-enter the supply chain, one company’s loss may be another’s gain: “[if company A] pushes out a demand for compensation from a supplier, the supplier will just tell them, no, and they’ll just continue to sell to [company B]. And [company B] knows that, ultimately, they’ll be forced to make the same commitment as [company A]. But that doesn’t stop them from dragging their feet, because they are going to financially benefit from that relationship, and are going to damage their competitor. And, and all of this work is being done for them by the campaigning NGOs, by the fact that they’re focusing on one company, rather than the sector” (CON-04). This leaves room for non-compliant companies to play buyers out against each other and try to negotiate supply chain exclusion and re-entry criteria in their own favor (CON-04, TRA-01).

### Responses to Paradoxical Tensions

In the face of the same paradoxical tensions, we can observe radically different responses by companies. We distinguish between constructive, prioritization and defensive responses and provide select examples below as well as in Table 10.

#### Constructive Approaches to Paradoxes

We identified constructive approaches—where companies use a ‘both/and’ mindset and acknowledge the tensions of demands while searching for creative approaches—most often in large, well-capitalized LISCs that are simultaneously highly exposed to Western markets and have a strong connection to the production base through owning their own mills and plantations. For such companies, full NDPE compliance is seen as a differentiating attribute: they are the ‘leaders’ that are competing for full goal attainment. For instance, while some companies have invested intensively in smallholder mapping to overcome the mentioned environmental-social paradoxes, others take a village-based approach to examining the deforestation risk in their supply sheds and work together with whole villages in the vicinity of their concessions on deforestation mitigation programs (TSO-02, TSO-05). Some companies create supply chain partnerships that aim to integrate mills and informal dealers as essential part of the supply chain, provide them with services that allow them to professionalize their operations, and otherwise provide ‘perks’ to incentivize their participation in supply chain sustainability efforts (LISC-05).

Such creative solutions come hand-in-hand with public explanations why the goal of 100% zero-deforestation had not been reached. Nestlé, for instance, was quoted as saying “we could have taken the option of removing smallholders from our supply chain but we did not,” with its board recognizing that “Nestlé could either keep smallholders and South America in its supply chain or meet its zero-deforestation pledge, but not both,” opting “for the slow and steady approach at the risk of appearing unreliable” (Chandrasekhar, 2019). Such transparency in general is welcomed by stakeholders, who note that “everybody who made an NDPE commitment almost had absolutely no way of just in a couple of years making sure that their supply chain was deforestation-free. So saying, who’s implemented it, and how successful have they been, is not about just saying how much of this product is deforestation-free, and how successful were they. It’s about how far have they got towards actually pulling the levers that need to be pulled to stop deforestation” (TSO-07).

#### Ignorance of Paradoxes or Prioritization of One Goal Over Another

Some companies decided to ignore tensions in order to reach their goal on time. However, this can backfire. A major consumer goods company and one of the few that self-declared to have reached their zero-deforestation goals was widely criticized and held up as a negative example for the way they achieved this goal—namely, by cutting their supply base from over 1000 mills to just over 100 highly vetted suppliers with low deforestation risk as their areas had been cleared before the cut-off dates (NGO-06, NGO-08, TSO-06, TSO-07). This action was seen to ignore the social aspect of smallholder inclusion, leading stakeholders to “raise concerns smaller farmers and suppliers that do not make the grade could be left behind” and protest that the company “needs to help more palm oil producers become sustainable rather than ditching those that do not meet its standards” (Taylor, 2020). One observer noted that “I think they’ve been guided maybe down a false path. […] They were convinced by Greenpeace in London that they just needed to focus on the D [for deforestation]” (NGO-06). By not simultaneously assessing demands for smallholder inclusion, this company did not achieve the perceived frontrunner status and related reputational improvements that come with private sustainability governance.

As another example of prioritization, a LISC representative said with regard to engaging with politically connected absentee farmers that “it’s not on the top of my priority list to be honest to engage. And, you know, we are happily moving other items on top of that” (LISC-05). This approach was justified by the impossibility of finding good solutions, and the political risk involved in doing so. However, such approaches risk leaving the most dangerous drivers of forest loss unaddressed in the near future.

#### Defensive Responses to Paradoxes and the Use of the State

Finally, we find a number of responses that can be categorized as defensive. Of particular interest are a version of what Pinkse et al. (2019, p. 328) call ‘destabilising’ responses where “companies introduce a competing tension to destabilise interventions or proposed measures […] by highlighting the unintended consequences or ineffectiveness of such measures.” For instance, a number of companies drew on an environmental-social sustainability tension framing to argue that further expansion in forested areas is necessary to help local communities develop. For instance, they argued “what happens if the community wants development and want to do it? [Should we say] ‘so sorry, we don’t want to touch you as long as you’re highlighted in the media’?” (GRO-02), or noted that “we believe that we have to respect orangutans. But then we have also orang asli, indigenous people, also to help” (GRO-03).

Civil society representatives, while recognizing local communities’ right to develop, countered that “that argument is not based on reality” (NGO-06) because previous palm oil expansion rarely improved local communities’ livelihoods and wellbeing. They further stressed that “almost by definition the local communities and indigenous peoples in high forest cover landscapes are going to be small in population. […] So it’s very important that […] the models that we are presenting aren’t just large-scale industrial oil palm because that means thousands of workers, intensive areas, so for the companies for who that is their model, it’s important to realize that their model will not work in those landscapes” (NGO-05), undermining the paradoxical tension constructed by large growers. NGOs also perceived that “smallholders are used as a loophole for [expletive] everyone” and that “it is that facade of ‘we are all committed’, but really, we are pushing for loopholes wherever we can make them, […] so they can maintain their corporate responsibility and can publicly report on progress” (NGO-06).

We also encountered perceptions that companies drew instrumentally on the legal compliance context in Indonesia to introduce new paradoxes and justify their inaction. Given its reliance on palm oil-related taxes and foreign currency, the Indonesian state has tended to make laws that facilitate plantation development (MSO-01, NGO-03, TSO-02). Companies risk having their permits revoked if they conserve forest on land zoned for agricultural development such as plantation concessions (Abandoned Land policy; Daemeter; Pacheco et al., 2020, GRO-02, LISC-01, TSO-03). State agencies have also warned growers against sharing information such as their concession maps with the wider public, citing competitiveness concerns (C.N.N. Indonesia, 2019, NGO-03, TRA-01), and threatened companies involved in collaborative action to achieve NDPE implementation with competition law enforcement (Dermawan & Hospes, 2018). Many companies referred to these restrictions to explain their lack of progress. Yet, some observers doubted that such legislation is a true binding criterion. For instance, with regard to the Abandoned Land policy, one expert explained that “they know that the regulation exists but they also know that there is no way that the government will enforce that. There is always a risk, of course. And so for certain companies in Kalimantan, I believe that they engage with their local government and set up the HCV area as a local conservation area” (NGO-03)—which would provide a pathway out of the bind presented by other companies.

Even more damningly, multiple stakeholders referred to the fact that select companies with sustainability commitments simultaneously lobbied the Indonesian government to pass decrees that make compliance more difficult, such as prohibitions on sharing concession information or threats to dissolve corporate collaborations. As one interviewee opined, “you’ve probably seen that the government of Indonesia lately came up with regulations about transparency of maps. How did the government of Indonesia come up with that? It is because of the lobby of these companies who are NDPE committed” (CON-05). In this way, corporate actors may create or exploit sustainability paradoxes in an attempt to justify limited action and a focus on more amenable, less stringent avenues toward corporate goals—at least in the eyes of external stakeholders.

### The Implications of Paradoxical Tensions for Goal Attainment

At the end of 2020, many observers noted with dismay the low goal attainment of companies with zero-deforestation commitments (CGF, 2021). Our analysis helps us understand that most companies failed to reach 100% deforestation-free supply chains due to grappling with performing and organizing paradoxes, whereas only a minority took an ‘either/or’ approach and pursued zero-deforestation supply chains irrespective of other goals. The range of responses to the same types of tensions can explain the differential progress that companies achieved on their path toward zero-deforestation commitment implementation (compare Table 11).

Yet, the most dominant companies in terms of volume implemented their commitments with an initial focus on their internal operations and large plantation companies, rather than their third-party and smallholder base. In addition, these types of companies have tended toward creative approaches due to the heightened attention on them and their competition for goal attainment leadership. In consequence, most companies agree that “deforestation from large scale plantation is trending down” (LISC-03), since growers understand that “if you want to grow greenfield [by deforesting], it’s essentially committing commercial suicide. Because, if you are deforesting, you will be blacklisted immediately and only with a very high cost will be able to come back on into the positive lists” (LISC-05). This impression is borne out by independent analysis, which finds that palm-driven deforestation in 2021 was at a 20-year low despite rapidly rising prices (Nusantara Atlas, 2022; see also the steady decline in palm-driven deforestation post-2013 in Fig. 1). While deforestation outside of concessions is an ongoing concern, the size of such clearings is (still) orders of magnitude smaller. Hence, until now, zero-deforestation commitments in the palm sector are seen to have contributed to the sizable decrease in palm-driven deforestation despite a lack of 100% achievement. It remains to be seen whether this attitude and corporate practices will persist.

## Discussion

### How Goal-Based Private Sustainability Governance Undermines Itself

Our case study suggests that goal-based governance is likely to give rise to paradoxical tensions. Our analysis identifies two main types of paradoxes: performing and organizing. Performing paradoxes arise from the competing goals of environmental and social and/or economic sustainability, with smallholder inclusion and grievance management being key issues. Organizing paradoxes arise between the choice to compete or cooperate on data sharing and grievance management. A number of companies also showed a strong proclivity to have defensive, or even destabilizing, responses that created new paradoxical tensions and undermined the proposed measures. We suggest that these three outcomes (the emergence of performing paradoxes, the emergence of organizing paradoxes, and the likelihood of destabilizing responses) are strongly linked to the characteristics of goal-based private sustainability governance.

Goal-based private governance foregrounds the importance of one, or a select few, goals, and builds enthusiasm and competition around reaching targets in those issue areas (McDermott et al., 2022). Goals are set as a result of civil society pressure and negotiation with businesses, rather than in an inclusive, multi-stakeholder setting (Lyons-White et al., 2020). This leads to a tendency to formulate absolute, clear, and snappy goals such as ‘zero deforestation’, which allows for little ambiguity or compromise at the point of goal setting. The ambiguity is instead relegated to the point of implementation, when the agreed-upon goals clash with social or economic realities, leading to *performing paradoxes*.

**Fig. 5:**
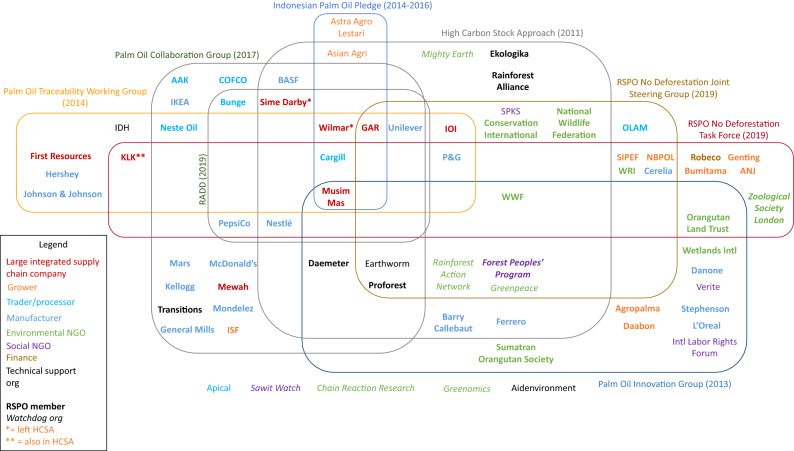
Key stakeholders in the zero-deforestation palm oil governance space, by membership of various multi-stakeholder groups. Own illustration

Performing paradoxes—particularly tensions between environmental, social, and economic goals—also exist in rule-based private governance, but such paradoxical tensions tend to be highlighted and discussed during the collective rule formulation process (Dentoni et al., 2018; Martens et al., 2017; Schouten et al., 2012). While contestation and deliberation may not completely resolve tensions (Bitzer & Schouten, 2022), it draws the collective attention toward harmonizing and aligning goals and their implementation steps, giving companies a clearer pathway to follow (Arenas et al., 2020). The lack of formal governance mechanisms in goal-based sustainability governance, in contrast, poses the danger that certain voices are underrepresented and remain unheard until the point of implementation. For instance, we can see in Fig. 5 (in the Appendix) that very few social NGOs participated in the various working groups to advocate on behalf of local communities or smallholder farmers.

Additionally, goal-based private governance is spurred by competition at the top. Civil society observers routinely construct rankings to distinguish between the best and worst performers, laud the best and shame the worst (Bartley & Child, 2014; Dubuisson-Quellier, 2021). Such rankings motivate companies to exert effort to move to the top. Yet, this approach also makes *organizing paradoxes* and a reluctance to cooperate more likely, as companies benefit from being ahead of their competitors in their implementation strategy. In rule-based private governance, in contrast, competition mainly occurs between members of the in-group (e.g., commodity roundtables) and the out-group, with the reputational advantages of membership being seen as a club good that benefits all members and induces cooperation in the in-group (Prakash & Potoski, 2006; Schuler, 2012). This approach may lower the likelihood of competition-cooperation paradoxes to the extent visible in goal-based governance—though Hahn and Pinkse (2014) theorize that organizing tensions between competition and effectiveness also exist in cross-sector partnerships.

Finally, companies pursuing private governance through goals have much greater leeway on the strategies they use to attain those goals, with companies’ legitimacy and performance being evaluated on to what extent they achieve their goal, rather than whether they adhere to collectively set rules. This set-up is similar to experimentalist governance which “accommodate[s] local diversity and foster[s] recursive learning from decentralized implementation” (Overdevest & Zeitlin, 2014, p. 22) in allowing for much greater learning and innovation in reorganizing practices toward sustainability (Búrca et al., 2014; Sabel & Zeitlin, 2008).

Yet, this flexibility is a two-edged sword: on the one hand, it brings forth creative solutions and genuine innovation in the way the industry has done things; on the other hand, companies that do not vie for leadership in goal attainment and have less to fear from reputational damage are enabled to hide behind recognized and manufactured paradoxes to justify that their commitments only exist on paper and not in practice. The existence of recognized performing paradoxes and companies’ struggles to deal with them empowers companies with little genuine interest in changing to pursue *destabilizing responses* that build on and play with the idea of paradoxical tensions, often using the state and its policies as reasoning why no change from business-as-usual is possible. Such destabilizing responses are also visible in companies arguing against a ratcheting up of collective rules during multi-stakeholder review processes, and may result in lower collective ambition of rule-based private governance (Dentoni et al., 2018). Still, at the multi-stakeholder level such discussions tend to be more transparent and the legitimacy of destabilizing responses can be easier checked by opposing viewpoints (Cheyns, 2014). The individuality of goal-based private governance strategies leads to a more complex picture where genuine and manufactured paradoxes coexist, overlap, and are a matter of interpretation by different stakeholders, leading to highly differential goal attainment.

### Practical Implications

Performing and organizing paradoxes are highly likely to occur anytime a relatively narrow, categorical collective goal is set. Unfortunately, many of the leading science-based targets that companies coalesce around, as well as a number of legislative initiatives such as the EU’s Regulation on Deforestation-Free Products (European Commission, 2021) fall into that category, which highlights the relevance of analyzing the implementation of such goals with sensitivity to potential tensions and trade-offs.

Besides this general insight, our paradox framing allows us to arrive at more context-specific policy recommendations. In cases where companies use paradoxical tensions strategically to postpone action, mandating greater action through legal means may be a way to raise the collective playing field. However, at other times, they face genuine limitations that they would equally encounter if they were legally mandated to implement supply chain due diligence. This is especially relevant because due diligence laws are most likely to be introduced at the downstream end of the chain (e.g., in Europe) (Bager et al., 2021).

Our results suggest that the sustainability community should actively try to reconcile key sustainability paradoxes that are being asked of ZDC firms by delineating best practices for constructive approaches between environmental and social sustainability goals, rather than inadvertently enabling firms to hide behind paradoxes and avoid implementing ZDCs by imposing simultaneous, irreconcilable demands. This is also urgently needed to avoid encouraging firms to prioritize effectiveness (via traceability and accountability) over equity (smallholder inclusion) in the competing sustainability demands, thereby harming already vulnerable populations of smallholder farmers (Grabs et al., 2021).

### Contributions and Future Research Directions

This article makes three major contributions to the private governance and business ethics literatures. First, it highlights through an in-depth empirical case study that the implementation of goal-based private sustainability governance is fundamentally distinct from the hitherto most commonly studied rule-based private governance. While much of the private governance literature has recognized the limitations of rule-based private governance via certification schemes and multi-stakeholder initiatives in bringing about sustained improvements in on-the-ground sustainability, the growing phenomenon of goal-based private governance has remained comparatively underexplored. As companies increasingly move private governance in-house and adopt future-oriented commitments, own-company standards, and supplier codes of conducts (Grabs, 2020; Thorlakson, 2018), it is of high relevance to better understand the company-internal and cross-company dynamics at play during the implementation phase of such approaches.

Second, our article gives insights into these dynamics by introducing the paradox perspective as theoretical lens through which to evaluate companies’ behavior. To our knowledge, this is the first instance that a paradox lens has been applied to an empirical case of private governance. By linking these two literatures, we show the analytical power of a perspective that is highly sensitive to company-internal dilemmas and that explicitly opens the’black box’ of the company while also considering companies’ interactions at a sectoral level. We encourage other authors to consider this perspective when assessing private governance implementation. This could be particularly helpful when trying to diagnose the potential causes of a lack of progress, as it allows us to move beyond a focus on greenwashing or contextual implementation challenges. In our case, it provides a more nuanced perspective of the real challenges that even good-faith actors face in implementing sustainability commitments and points to a need for policy makers and civil society to refine their demands on the private sector (see Sect. “Practical Implications”). However, we also show that companies’ responses to paradoxes in practice range from constructive to defensive, and may undermine the theory of change of goal-based private governance in important ways.

Third, our case study provides interesting takeaways for paradox theory. It underscores the importance of furthering research into nested, knotted, and interwoven tensions in which the management of one paradoxical tension creates another paradox (see the opening quote for a clear example) (Waldner et al., 2022). Such nested tensions may be particularly prevalent in the context of wicked problems—issues with knowledge uncertainty, dynamic complexity, and value conflicts among stakeholders –, for which palm oil sustainability is a good example (Dentoni et al., 2018). Our focus on how various companies manage paradoxes further provides indicative evidence that higher-resourced companies with a greater reputational risk tend to be more aware of paradoxes and take more creative paths to try to manage them, which tends to create improved impacts on the ground. We encourage other paradox theory researchers to move from single company case study approaches to more holistic sectoral or cross-sectoral assessments and systematically assess which types of companies pursue which types of responses, as well as how different approaches culminate in actual impacts on the ground.

We recognize the limitations of our study, such as our reliance on information from organizations willing to talk to us, as noted in the Methods. In addition, our insights derive from examining goal-based private governance implementation in a single sector. While we hypothesize that the insights presented are indicative of developments in other types of goal-based private sustainability governance, this hypothesis should be tested in future research. We anticipate that the results hold particularly in similar contexts such as goal-based governance in agricultural commodity sectors. For instance, the cocoa supply base is made up almost entirely of smallholders, raising the issue of how to assure traceability and deforestation prevention (in the context of the Cocoa and Forests Initiative) while not excluding smallholder farmers or jeopardizing companies’ supply bases (Carodenuto, 2019). The cattle sector has experienced similar struggles over whether to include indirect suppliers into zero-deforestation commitments as the palm oil sector. Even beyond the environmental agenda, social sustainability goals such as eliminating child labor, forced labor, and modern slavery likely raise similar challenges (LeBaron, 2021). Future work may find it fruitful to employ the presented theoretical approach to analyze goal-based private governance in these areas.

Our research also opens up a number of additional future research avenues. On the topic of organizing tensions between cooperation and competition, the ‘co-opetition’ literature studies in greater detail how companies navigate such tensions (Munten et al., 2021; Stadtler, 2018; Stadtler & Van Wassenhove, 2016). While going beyond the scope of this article, it would be an interesting future research question to conduct a dynamic analysis of how companies choose and switch between cooperation and competition-focused company-internal innovation when pursuing the implementation of goal-based sustainability governance. The role of competition law in constraining collective sustainability efforts is an equally promising research area that has been relatively understudied in the sustainability governance literature, though it has received incipient attention in the business ethics and legal disciplines (Claassen & Gerbrandy, 2018; Dubbink & van der Putten, 2008).

A further research direction constitutes the progressive institutionalization of goal-based private sustainability governance (cf. Grabs, 2020). While there is still no one pathway toward verifying zero-deforestation palm oil, the uncertainty and duplication of efforts outlined above have led industry members in various forums (including the Roundtable on Sustainable Palm Oil) to work towards standardization and codification of best practices. As this process is not yet concluded, it appears too early to comment on its success. However, it is possible that goal-based private sustainability governance as characterized above is a primarily short-term phenomenon that is drawn upon to socialize new ideas that are then progressively integrated into existing regulatory structures. The interaction between goal-based and rule-based governance, especially in the same sector, is therefore an interesting avenue for further theorization.

## Conclusion

Many companies with zero-deforestation commitments aimed to eliminate deforestation from their supply chains by 2020. Yet, barely any company reached that target (CGF, 2021). Our article uses the case study of zero-deforestation palm oil to argue that this achievement gap can be explained by paradoxes that arise in goal-based private sustainability governance.

We argue that the characteristics of goal-based private sustainability governance—a collective, but non-inclusive goal-setting process; goals that may be perceived as binding in case of high reputational risk; companies enjoying extensive leeway in implementing goals; and actors relying on weak institutional arrangements to align practices—makes goal-based private sustainability governance particularly susceptible to the likelihood of arising performing paradoxes (related to contradictory demands and goals) and organizing paradoxes (related to ways in which companies act; here specifically related to cooperation versus competition). Of particular concern in the case study are environmental-social sustainability paradoxes related to ensuring the inclusion of smallholders and more marginalized producers in zero-deforestation value chains. We also found that companies perceive paradoxical tensions in delivering greater accountability for deforestation events while maintaining a competitive supply base; and in transforming industry-wide problems through collective action while maintaining a competitive edge and respecting anti-trust law. As a result, firms resort to a wide range of responses that imply vastly different sustainability consequences. While creative approaches may ultimate help resolve emerging paradoxes, defensive approaches and the ignorance of tensions stand to further undermine progress toward zero-deforestation supply chains.

Ultimately, our study highlights the overarching paradox behind goal-based private sustainability governance: While ambitious goals may be useful to spur innovation and industry rethinking, it might be preferable to accept limitations in reaching such goals due to goal tensions until pathways are found that avoid trade-offs. Yet, as consuming regions take more responsibility for their contributions to global sustainability challenges, it remains urgent to revisit how changes in behavior and policies in these regions and associated supply chains can contribute to reconciling the underlying tensions of trying to achieve sustainable supply chains, for example, by more directly addressing over-consumption and unequal power distribution.

## Data Availability

Full, anonymized transcripts are available from the authors upon request.

## Appendix

Fig. 5, Tables 4, 5, 6, 7, 8, 9, 10, 11

**Table 4:** Overview of workshops attended during data collection

Date	Location	Event organizer	Event name	Link	Code
04.03.20	Jakarta	UKRI GCRF TRADE Hub	UKRI GCRF TRADE Hub Research Workshop	N/A	FN-01
15.05.20	Virtual	Chain Reaction Research	Which companies are most exposed to deforestation-driven fires in their supply chains?	https://chainreactionresearch.com/event/crr-webinar-which-companies-are-most-exposed-to-deforestation-driven-fires-in-their-supply-chains/	FN-02
03.06.20	Virtual	Earthworm	Deforestation-free palm oil supply chains: how are companies progressing towards meeting their 2020 target?	https://www.youtube.com/watch?v=TTzqCExm1ME&feature=youtu.be	FN-03
04.06.20	Virtual	RSPO	RSPO Jurisdictional Approach Public Consultation	N/A	FN-04
10.06.20	Virtual	GIZ	BMZ Virtual Forum on EU-Measures for Deforestation-Free Supply Chains	https://www.nachhaltige-agrarlieferketten.org/en/news-events/bmzvirtualforaoneu-measuresfordeforestation-freesupplychains/?no_cache=1?no_cache=1?no_cache=1?no_cache=1	FN-05
24.06.20	Virtual	RSPO	RSPO North America Sustainable Palm Oil Dialogue: Deforestation and the Downstream from Policies to Implementation	https://www.youtube.com/watch?v=Txkh0CA4lGQ&feature=youtu.be	FN-06
24.07.20	Virtual	Tropical Forest Alliance	TFA Dialogue: Raising Ambition for Effective EU Policy to Protect the World’s Forests	https://www.youtube.com/watch?v=RgKh4v_ww7Y&feature=youtu.be	FN-07
02.09.20	Virtual	European Palm Oil Alliance, RSPO, IDH	Europe Sustainable Palm Oil Dialogue	N/A	FN-08
10.09.20	Virtual	GeoAwesomeness	Enabling sustainable supply chains through satellite technology	https://www.geoawesomeness.com/2020-geoawesomeness-digital-meetup-schedule/	FN-09
03.–05.11.20	Virtual	Innovation Forum	Sustainable Landscapes and Commodities Forum	N/A	FN-10
09.11.20	Virtual	Chain Reaction Research	FMCGs’ Zero-Deforestation Challenges & Growing Exposure to Reputation Risk	https://chainreactionresearch.com/event/webinar-fmcgs-zero-deforestation-challenges-growing-exposure-to-reputation-risk/	FN-11
25.11.20	Virtual	CDP	Achieving a resilient and sustainable palm oil supply chain through private—public collaboration for sustainable development in Indonesia	N/A	FN-12

**Table 5:** Overview of interviews conducted during data collection

Nr	Date	Location	Organization type	Interviewee role(s) [abridged or edited to preserve anonymity]		Interview length	Interview use	Code
1	17.09.19	Virtual	NGO	Deputy director		35 min	Informational	N/A
2	18.09.19	Virtual	RES	Assistant professor		44 min	Informational	N/A
3	20.09.19	Virtual	NGO	Director		44 min	Informational	N/A
4	30.09.19	Virtual	GRO	Plasma support manager		38 min	Informational	N/A
5	01.10.19	Virtual	NGO	President		31 min	Informational	N/A
6	01.10.19	Virtual	NGO	Senior analyst		31 min	Informational	N/A
7	04.10.19	Virtual	TSO	Senior program officer		48 min	Informational	N/A
8	07.10.19	Virtual	NGO	Technical advisor		40 min	Informational	N/A
9	10.10.19	Virtual	RES	PhD student		50 min	Informational	N/A
10	11.10.19	Virtual	TSO	Senior program officer		48 min	Informational	N/A
11	18.10.19	Virtual	RES	Lecturer		28 min	Informational	N/A
12	15.11.19	Virtual	TSO	Program manager		41 min	Informational	N/A
13	13.12.19	Switzerland	TSO	Project manager		60 min	Informational	N/A
14	28.01.20	Switzerland	NGO	Senior program manager		70 min	Informational	N/A
15	19.02.20	Virtual	MSO	Associate manager		42 min	Informational	N/A
16	19.02.20	Virtual	RES	Assistant professor		38 min	Informational	N/A
17	21.02.20	Virtual	NGO	Global palm oil lead		52 min	Coded	NGO-01
18	27.02.20	Indonesia	DEV	Project leader; consultant		75 min	Coded	DEV-01
19	28.02.20	Indonesia	TSO	Senior program manager; program officer		95 min	Coded	TSO-01
20	01.03.20	Indonesia	CON	Consultant		79 min	Coded	CON-01
21	02.03.20	Indonesia	MSO	Director of operations; senior manager		61 min	Coded	MSO-01
22	02.03.20	Indonesia	CON	Consultants		60 min	Coded	CON-02
23	02.03.20	Indonesia	NGO	Project lead; research analyst		91 min	Coded	NGO-02
24	03.03.20	Virtual	DEV	Project coordinator		63 min	Coded	DEV-02
25	03.03.20	Indonesia	TSO	Project manager; manager		76 min	Coded	TSO-02
26	04.03.20	Indonesia	GRO	Vice president of sustainability		35 min	Coded	GRO-01
27	05.03.20	Indonesia	NGO	Sustainable commodities manager		104 min	Coded	NGO-03
28	05.03.20	Indonesia	MSO	Associate manager		176 min	Informational	N/A
29	10.03.20	Indonesia	SHO	Coordinator		61 min	Coded	SHO-01
30	10.03.20	Indonesia	GRO	Chief Operating Officer		75 min	Coded	GRO-02
31	11.03.20	Indonesia	GRO	Sustainability director, sustainability advisor		81 min	Coded	GRO-03
32	15.03.20	Indonesia	TSO	Regional manager		85 min	Coded	TSO-03
33	17.03.20	Virtual	SHO	Coordinator		54 min	Coded	SHO-02
34	19.03.20	Virtual	TSO	Deputy director		73 min	Coded	TSO-04
35	19.03.20	Virtual	NGO	Project manager		86 min	Coded	NGO-04
36	20.03.20	Virtual	CON	Country director		41 min	Coded	CON-03
37	23.03.20	Virtual	GRO	Head of investor relations		74 min	Coded	GRO-04
38	25.03.20	Virtual	TSO	Operations lead		59 min	Coded	TSO-05
39	30.03.20	Virtual	NGO	Policy advisor		32 min	Coded	NGO-05
40	08.04.20	Virtual	LISC	Vice president of sustainability		53 min	Coded	LISC-01
41	10.04.20	Virtual	LISC	General manager, sustainability		48 min	Coded	LISC-02
42	14.04.20	Virtual	TRA	Sustainability leader		55 min	Coded	TRA-01
43	21.04.20	Virtual	GRO	Sustainability advisor		49 min	Coded	GRO-05
44	23.04.20	Virtual	TRA	Stakeholder relations manager		87 min	Coded	TRA-02
45	24.04.20	Virtual	CON	Chief sustainability officer		52 min	Coded	CON-04
46	24.04.20	Virtual	LISC	Head of policy		48 min	Coded	LISC-03
47	18.05.20	Virtual	LISC	Head of sustainability		60 min	Coded	LISC-04
48	30.07.20	Virtual	TRA	Global sustainability lead		53 min	Coded	TRA-03
49	11.09.20	Virtual	CGM	Sustainable agriculture manager		56 min	Coded	CGM-01
50	12.11.20	Virtual	LISC	Head of sustainability		50 min	Coded	LISC-05
51	18.11.20	Virtual	LISC	Head of sustainability		59 min	Coded	LISC-06
52	19.11.20	Virtual	CON	Strategic sustainability advisor		57 min	Coded	CON-05
53	24.11.20	Virtual	CGM	Global sustainability lead		43 min	Coded	CGM-02
54	25.11.20	Virtual	TRA	Sustainability manager		63 min	Coded	TRA-04
55	30.11.20	Virtual	TSO	Head of client relations		52 min	Coded	TSO-06
56	02.12.20	Virtual	NGO	Policy director		61 min	Coded	NGO-06
57	04.12.20	Virtual	CGM	Sustainability lead		51 min	Coded	CGM-03
58	04.12.20	Virtual	TSO	Consultant		55 min	Coded	TSO-07
59	07.12.20	Virtual	NGO	Executive director		58 min	Coded	NGO-07
60	16.12.20	Virtual	CGM	Sustainable oils manager		52 min	Coded	CGM-04
61	17.12.20	Virtual	CGM	Global sustainability lead		50 min	Coded	CGM-05
62	18.01.21	Virtual	NGO	Program director		59 min	Coded	NGO-08
63	29.01.21	Virtual	TSO	Program lead		58 min	Coded	TSO-08
CGM: Consumer goods manufacturer CON: Consultant DEV: Development organization GRO: Grower LISC: Large integrated supply chain company MSO: Multi-stakeholder organization					NGO: Environmental and/or social NGO RES: Researcher SHO: Smallholder organization/representation TRA: Trader/processor TSO: Technical support organization

**Table 6:** Overview of challenges related to deforestation in the Indonesian oil palm sector

Deforestation-related challenges	Large-scale concessions	Medium- and small-scale actors
Past deforestation and/or development in Forest Estate and protected areas	Current legality of plantations Contentious land rights (esp. regarding indigenous and customary lands) Compliance with ZDC cut-off date	
	Rules on roundtable membership (e.g., RSPO) and supply chain access for companies that deforested after cut-off (incl. mergers and acquisitions) Compensation and legacy cases	Livelihood alternatives for current producers of illegal palm oil
Current deforestation	Legal alignment and follow-through on conservation set-asides Identification of deforestation on concession Use of market power to enforce stop-work orders Extension of oversight to third-party (indirect) suppliers	Traceability to medium- and small-scale plantations Dissemination of ZDC criteria among small-scale actors Tracking current deforestation and linking it to future supply chain (3–5 year time lag) Inclusive sustainable supply chains
Future deforestation	Closing of leakage market Lowering the financial incentive for further concessions/ conversion Monetization of stranded assets	Prevention of expansion in response to ageing plantations Prevention of replanting in current illegal areas and development of alternative options Balancing right to development of forest-dwelling communities wishing to be integrated in global economy

**Table 7:** Evidence for characteristics of goal-based private sustainability governance

Characteristics of goal-based private sustainability governance	Exemplary evidence
Goal-setting process: Goal-setting process is collective, but not inclusive, as it is dominated by negotiations between leading NGOs and corporate actors	“The whole palm industry has changed a lot in 2015 as the first company, Wilmar, that actually declared on the NDPE commitments and it actually influenced a lot of the other supply chain players. […] But no matter how big or how small is the volume, the expectation of the stakeholders on the palm industry is quite standardized, so we have followed through in terms of how we update the commitments.” (LISC-02) “I was doing a range of activities like helping them at the beginning formulate NDPE policies, benchmark NDPE policies and helping them develop commitments, aligned to their own company vision that were competitive within the market.” (CGM-03) “NDPE wasn’t even a concept more than around three years ago. […] Wilmar was the biggest commitment that came in early 2014. So in just about six years, that has moved from being something that was unheard off to being something which is really an expectation and market basis, […] I don’t think you’ll find any of the big international companies buying from companies that don’t have NDPE commitments. So it’s become an industry standard, really, I think for at least the export orientated companies.” (CGM-01) “Ultimately, they went after one big player, and that was Wilmar. And you know, it is essentially like the Walmart for palm oil, like every single supplier and buyer goes to them at some point. So once they started setting rules, then the market started responding.” (CON-03)
Binding or non-binding: Perceived binding-ness of goals depends on reputational risk and stakeholder evaluations	“Well, it has changed because before we never made a promise, to stakeholders, to the public, before. Now we have made a public promise and people can ask ‘well what about your promise’ if you don’t follow it.” (GRO-01) “To be honest, I think it has taken time for people to really understand that essentially greenfield growth in Indonesia is over, if you want to grow greenfield, it’s essentially committing commercial suicide. Because, if you are deforesting, you will be blacklisted immediately and only with a very high cost will be able to come back on into the positive lists, right […] In Indonesia, companies made a lot of commitments, and then they just ignore it. And somehow I think they realized, so this time, we don’t get away with ignoring it anymore. So this time we really need to implement and it has taken a lot of time to realize that.” (LISC-05) “Everybody who made an NDPE commitment almost had absolutely no way of just in a couple of years making sure that their supply chain was deforestation free. So saying, who’s implemented it, and how successful have they been? It’s not about just saying how much of this product is deforestation-free, and how successful were they. It’s about how far have they got towards actually pulling the levers that need to be pulled to stop deforestation.” (TSO-07) “There’s quite a large number of upstream companies that now have NDPE policies in place. And in the beginning, they didn’t do a lot, or they implemented it on paper. But there was not a lot of action related to it. Yeah, there were, of course, reasons for that, one of them is no monitoring, and things like that.” (TSO-06)
Amount of leeway for implementation: Companies enjoy extensive leeway in implementing goals, though subject to stakeholder evaluation and progressive alignment	“NDPE [No Deforestation, No Peat, No Exploitation] is just a word. It’s a slogan, it doesn’t have any protocols, no indicators, no principles and criteria. But RSPO [The Roundtable on Sustainable Palm Oil] has got that. And NDPE is a famous buzzword where everybody advocates NDPE. And that’s why people follow it is just three sentences that’s it. But there’s so much clout in there.” (GRO-02) “And that also comes back in what we see related to the zero deforestation 2020 targets. These targets were set. But companies had no idea how to accomplish it. And then a year ago, we started receiving questions like Yeah, but how are we actually going to measure that? So companies set the targets, they had no idea how they were going to do it. And now really is time that they start to think about, okay, how are we going to measure, implement and monitor? It’s pretty interesting.” (TSO-06) “You’ve got another issue, which is, we are competing, and there is no real standout on how you demonstrate NDPE compliance or the virtue of your supply base.” (LISC-04)
Institutional arrangements: Reliance on weak institutional arrangements	“So what has NDPE done, the NDPE collaboration basically and there’s a collaboration group actually active it’s called the palm oil collaboration group, hosted and facilitated by PepsiCo, Cargill, and Proforest, you might have heard of that. And basically, that group has been jointly working on a harmonized way of reporting but also driving progress.” (CGM-04) “It’s pretty informal, I don’t know that there’s a formal setup or anything like that. And it doesn’t have a governance structure or anything like that. It’s just sort of “Let’s come together and try and tackle some of these [issues].” But you’ll see that there’s quite a good representation of, of different industry representatives, and you’ve got, you’ve got your campaigners, and your service providers and such like that, that are there and have attended the meetings and such like, so it’s pretty broad.” (CGM-01) “Over the past six years, we’ve seen unprecedented collaboration across the industry. Competitors, peers, suppliers, customers working together that would have never happened beforehand.” (TRA-01) “So many of the issues in palm are not supply chain issues, but systemic issues. And so you can only really address them at the industry level. And in many cases, not even that, eventually it needs others like government and such like to get involved. And so that’s where we have to play our part as sort of shared responsibility approach that we need to be able to lead the way if we say that this is, this needs industry change, and we ought to be there in the industry trying to encourage that change. And that’s why we’re involved in a lot of these groups or are convening a lot of these kind of initiatives.” (CGM-01)

**Table 8:** Themes describing informants’ experience of issues and approaches to solutions following Smith (2014)

Theme	Language used	Illustrative quotes
(Paradoxical) tensions 19 interviews, 38 references	Tensions, yet, but, however, balance, on one hand/on the other hand	“There are **tensions**, push and pull factors within large companies. With some teams pulling in one direction, and some in another. […] So yeah, those tensions certainly exist, as they probably should, within a healthy company that needs to be dynamic, it needs to have different teams focused on different priorities. And in the end, the **balance** should keep the company successful commercially, **but also** hopefully moving in the right direction, in terms of addressing social and environmental issues.” (CGM-03) “We protect forest from deforestation. Yes. But **on the other hand**, this palm-based economy has become a strong economic source so it’s not that easy to… So do we need to keep going with this zero deforestation issue even for smallholders? Yes, but maybe, I mean, it’s not a one or two year of works to bring all smallholders into sustainable zero deforestation practice. It takes many aspects that we must deal with so we can **balance** this rural economy and zero deforestation.” (NGO-02) “And it’s a **delicate balance** because NGOs, we don’t want to name-and-shame buyers, [but want to point out that they] are never going to meet their deforestation-free commitments until we come up with a solution with the third-party supply. **However**, we really support the fact that they published the mills, which allows us to piece this puzzle together, and we encourage them to stay involved in these landscapes to help resolve the problems.” (NGO-01) “It’s a very **delicate balance**. You have the amnesty for now, **but** we will also prepare a severe punishment if this happens in the future. But to send these two signals together is a **tricky** thing to do.” (NGO-03) “We’re trying to **strike a balance**, where we’ve understood the problem [and] see whether there’s any avenue we can bring to a **compromise** rather than just stopping.” (GRO-02) “So you need to look at what our sourcing teams wants, when our commercial team wants to deal with the mills, we need to look at whether we are so stringent that—you get what I mean, we have to **balance** this sustainability and business as well.” (TRA-02) “You cannot have both [no deforestation and smallholder inclusion], you can have one, you can have the other. And if you want to have both, you have to put some skin in the game and say, I will support change, and it will cost me. The problem is, if your neighbor doesn’t do it. Well, your marketing team is going to say, why do we do that? We’re going to get hit and we’re going to lose market shares. It’s an **uncomfortable balance to find**.” (LISC-04) “**Even** when companies try and monetize their sustainability, to try and **balanc**e the costs by going through green bonds, or even grey bonds and then in the social or even sustainable development bonds, everybody comes out of the woodwork, crying, greenwash foul.” (LISC-06) “Commercial and sustainability, I think **they don’t really go hand in hand**. But we are trying to **make them go hand in hand somehow**. And our company policy is very clear cut. It’s like we [want to do] ESG, we want to achieve some progressing on ESG. So, yeah, ultimately we will try to make this two parts of the business work.” (TRA-04) “Palm is an emotive material, but it has this **dilemma**. It is **on the one hand** the most productive vegetable oil. It is present in half of the products in every supermarket. **But** it is driving mass deforestation. So do you stay with palm or get out of it. We decided to stay, **but** make it truly sustainable.” (FN-10) “There are always pros and cons dealing with a big-sized company like that. The pros is of course they have significant buy-in power and also large areas of operation. So they can easily influence the behavior of the smallholders. If you therefore [have a] success approach that also serves their interest, they can easily replicate [it] to many other areas. But the negative side of working with them is that they have **very complex decision-making**. At the moment I am dealing with a second level after the chairman, and still this guy cannot control the decision. So you really have to go to the number 1 person. And there is conflict of interest among the second layer and it looks likely that the first person—maybe he knows and maybe it’s also his design that there is **no interest for him to mediate or resolve the different interests**.” [DEV-01]
Either/or dilemmas 5 interviews, 5 references	Tradeoffs, choice, resolve, either/or	“How do we avoid consumer outrage moving into more noise, more polarization, more division, as opposed to a more thoughtful conversation about the **trade-offs** between environmental protection and rural development. Those are **trade-offs** our companies have to deal with every day.” (FN-10) “It’s better just work together and **resolve** the problem, then we’re all happy.” (TSO-03)

**Table 9:** List of key (paradoxical) tensions between various stakeholder demands/logics

Issue	Description of issue and (paradoxical) tensions	Tension between…	And…	Illustrative quote(s)
*Achieving full supply chain traceability and transparency*				
Establishing more simple and direct supply chains 19 interviews, 32 references	Tension: To improve traceability, buyers may aim to streamline their supply (reduce the number of suppliers) and connect producers more directly to markets (reduce the number of intermediaries). This threatens to exclude more messy or informal parts of the supply chain and threaten the livelihoods and business models of intermediaries (agents) who may have powerful statuses in the community and/or provide important secondary services such as financing.	Environmental sustainability	Social sustainability	“Dealers play a really vital role in the supply chain because they provide financing, they provide logistics, sometimes they also provide technical assistance to these farmers sometimes. So again, they’re key players in the supply chain and you can’t just get rid of them because they have established these supply chains for a very long time and these communities rely on them.” (TSO-05)
Sharing identity of suppliers for traceability 14 interviews, 27 references	Paradox: Full traceability requires knowing the identity of upstream supplier tiers. Only first-tier suppliers or intermediaries usually have this information. Yet, providing it may endanger their business model, making actors reluctant to pass information along. Yet, if they do not provide traceability, actors along the supply chain may face negative business consequences.	Vertical cooperation	Competition	“Mapping our whole supply chain has been a five year project. That’s where even just getting all our suppliers to tell us what mills they source from has been real[ly difficult]. There’s so much commercial information in there, if we know the mill, we can cut our supplier out of the supply chain or vice versa.” (LISC-02)
Sharing supply base maps 11 interviews, 14 references	Paradox: To ensure accountability and verify grievances, civil society groups have pushed for corporate groups to share the concession boundaries of their suppliers. Yet, sharing such information may erode trust and make it more difficult for supply chain actors to achieve traceability. The Indonesian government further instructs palm oil businesses to avoid sharing such information. This, of course, undermines the efficacy of the accountability mechanism, which may have ramifications for the market access of all players involved.	Environmental sustainability	Economic sustainability *[reinforced by public policy]*	“You can imagine why companies are also hesitant, even if they are allowed to share concession data, about sharing it, because they don’t have the control over how it’s going to be used.” (TRA-01) “Map is a little bit like you’re in my personal data, like home address and bank account, right? You don’t want this abused.” (LISC-03) “There is no actual law that prohibits that. So the one that is being cited [is] a circular memo to all holders of palm oil licenses to not disclose any information to third parties including auditors. Which is ridiculous. It is a circular memo. The public information disclosure act is a law. It’s much much higher than this circular memo. But the memo is enough as a political gesture for these companies to not want to disclose. They will cite that ‘oh, the Coordinating Ministry of Economic Affairs prohibits me from sharing the boundary’.” (NGO-03)
*Maintaining smallholder inclusion*				
Including smallholders in traceability efforts 33 interviews, 74 references	Tension: Traceability of deforestation-free palm oil requires full visibility of the plantation supply base, including plot boundaries and ownership. However, the independent smallholder supply base is dynamic and informal, with suppliers likely to change on a regular basis, making full visibility cost-prohibitive.	Environmental sustainability	Social sustainability	“Unless we have a [GPS] coordinate for the smallholders, [our buyers] are saying, oh, it doesn’t meet our policy, and so you have to change or else we risk losing our whole business.” (TRA-03)
Supporting compliance of smallholders and mills 29 interviews, 66 references	Paradox: 40% of Indonesian palm oil production land is managed by smallholder farmers, who tend to lack the legal resources, technical and financial capacity to comply with supply chain policies. Since each committed company may have millions of smallholder farmers in their supply base who may switch back and forth, providing comprehensive compliance support may be highly resource-intensive. However, both excluding smallholder farmers and allowing for smallholder-based compliance exceptions runs against stakeholder expectations and may create reputational risks. If smallholders are excluded, this may also undermine the company’s impact on decreasing deforestation driven outside of concessions.	Environmental sustainability	Social sustainability and Economic sustainability	“You cannot have both [no deforestation and smallholder inclusion], you can have one, you can have the other. And if you want to have both, you have to put some skin in the game and say, I will support change, and it will cost me.” (LISC-04) “I think there’s an absolute tension between the smallholder engagement versus no deforestation commitments. I think the companies are aware of that.” (TSO-07) “Right now, no matter how green certain companies look like, they don’t want to pay. So these people [smallholders] have to pay.” (CON-05)
Preventing deforestation outside of concessions 17 interviews, 35 references	Paradox: Deforestation increasingly occurs outside of large-scale concessions. The responsibility for such clearing is empirically difficult to verify and is alternately attributed to smallholders, or to mid-scale actors (land barons) that hide behind the smallholder label for impunity. Supporting smallholder exceptions when enforcing commitments may provide accountability loopholes for such mid-scale actors. Yet, being more stringent on punishing deforestation outside of concessions may push such actors into leakage markets while decreasing companies’ influence over smallholder practices.	Environmental sustainability	Social sustainability	“I remember the same debates on smallholders, whether we’re expecting small farmers to actually uphold the principles and criteria, and how a kind of more lenient [approach] without losing sight of principles should be adopted. And how many of the hardliners said no.” (LISC-06) “Yes, it is of concern that NDPE policies could result in the exclusion of smallholders. But it’s also of concern that smallholders are used as a loophole for fucking everyone. And I think that that’s where the industry [actors] are currently positioning.” (NGO-06) “They have such a lean cost structure that they can sell wherever without going through our mills, for sure, our refineries, and so it’s so these are the guys who you are, you are losing them very, very quickly. As soon as you are putting a bit pressure on them, you are losing that supplier.” (LISC-05)
Supporting community development (especially HFCC) 16 interviews, 41 references	Paradox: Local communities, especially in high-forest cover countries or areas that have missed out on development in the past, may be interested in local development opportunities through palm oil planting. Strict enforcement of ZDCs would exclude the resulting palm oil from sustainable markets. However, in the absence of sustainability rules, other income-generating activities (e.g., charcoal) may drive deforestation.	Environmental sustainability	Social sustainability	“I guess it’s a dilemmatic situation for the company as well as for the government, because agriculture still remains our priority economic development. So we rely on that. So when we want to conserve forest areas, that means we cannot develop them, which means it doesn’t bring any economic development impact to the local community.” (TSO-02) “We believe that we have to respect orang utan. But then we have also orang asli, indigenous people, to help.” (GRO-03) “And that was quite brutal in terms of having to tell people who actually are relying on agricultural development [that] we couldn’t develop. […] It’s very hard for people to understand that these rules apply when it’s their forests.” (LISC-06)
Responding to smallholder illegality 15 interviews, 31 references	Paradox: A number of smallholder palm areas lie in national parks or are otherwise illegal. As companies uncover their links to such producers through traceability improvements, they face tensions between supporting the livelihoods of smallholders in their supply chains, or excluding them to respect local laws and ZDC pledges. If they exclude them, however, they may lose influence over farmers that continue to clear.	Environmental sustainability *[reinforced by public policy]*	Social sustainability	“And for the farmers and independent farmers, their main subsistence, livelihood is mainly from that. If finally we decide that we’re going to stop this purchase it will be complete chaos and mayhem. They may even burn down our factory because you’re completely isolating them. You’re completely destroying their livelihood, you are completely destroying this community as a whole.” (GRO-02) “But some farmers, you will not be able to get their legal license, because of the locations where they are. So then you need to think about okay. But today they earn money. So are we going to take it away? Or can we offer them alternatives. […] And it’s merging the two key objectives of the Sustainable Development Goals, I think. So it’s indeed lifting out of poverty versus protecting biodiversity.” (CGM-04)
*Defining, incentivizing, and enforcing compliance*				
Defining deforestation 12 interviews, 22 references	Tension: Deforestation needs to be clearly defined for compliance enforcement to take place. But agreed-on definitions (e.g., HCSA) are in conflict with Indonesian government definitions and do not (yet) provide clear guidelines or adaptations for smallholders, which might contribute to their exclusion. This may make ZDC enforcement challenging.	Environmental sustainability	Social sustainability *[reinforced by public policy]*	“If you ask the Ministry of Agriculture or the district or provincial office for estate crops, they will say we don’t have any deforestation. Because in their dictionary deforestation means [that] a company or person opens forest area without any permit.” (NGO-04)
Shaping of market expectations in growth markets 18 interviews, 36 references	Tension: The lack of demand for sustainable products in growth markets opens up leakage markets and opportunities for non-committed actors to sell deforestation-associated palm oil. Adhering to ZDCs lowers the competitiveness of committed actors to supply those same markets. Yet, disregarding ZDCs is risky as it is unclear whether dominant market actors in such markets might change their expectations in the future.	Horizontal cooperation	Competition	“And the fact is, is that they’ll never have a level playing field with the leakage market; the people who actually just choose to ignore these commitments are still sort of winning.” (CON-04) “And we are so scared that the European market will influence the China market, Indian market. It’s already happening. Yeah, basically now Japan, Korea. They already require sustainability. China also.” (GRO-03)
Providing commercial incentives for deforestation-free production 41 interviews, 138 references	Paradox: Growers incur compliance and opportunity costs in identifying and protecting forest land in plantation areas. Commercial incentives could increase engagement and compliance, but increase costs of midstream and downstream buyers. On the other hand, a failure to provide commercial incentives may result in more deforestation events and reputational risks for buyers.	Vertical cooperation	Competition	“Because there are markets out there that still allow these companies to send their fruit for another market, in [the NDPE] market it is still a big question mark of how do you drive sustainability and the incentive mechanisms are not yet there.” (TSO-05) “I know it’s something that’s encouraged by the NGOs to provide financial incentivization to suppliers to be acting on the up and up, but we have that as the basis of requirement for doing business. Our expectation is that suppliers are going to be acting in an ethical and responsible way and that they’re going to be responsibly sourcing their product for us.” (CGM-05)
Monetizing stranded assets and legacy cases 15 interviews, 38 references	Paradox: Companies that invested in concessions and infrastructure before ZDCs were adopted now face high write-offs if such areas cannot be developed (‘stranded assets of high value’). To avoid exploitation, such assets might be otherwise monetized, e.g., through carbon credits. Yet, such schemes may face legal hurdles (regarding changing license status). If companies do not monetize them, they may face bankruptcy; but if they clear, market exclusion and restricted earnings, making environmental sustainability a precondition for sustained economic sustainability. The third option—creating exemptions for legacy cases—de facto would allow for more palm-driven deforestation to take place and is strongly opposed by environmental interest groups.	Environmental sustainability	Economic sustainability *[reinforced by public policy]*	“The timing is all different. By the time RSPO adopted NDPE style requirements, some of us have already got concessions in high forest cover landscapes. […] So they will need to create conditions for legacy members who have got this high forest cover landscapes and what to do with them otherwise it will become a stranded asset. […] The predicament we faced was not completely our fault. We acquired the concessions long before even NDPE was born.” (GRO-02) “The more cash strapped the business is, the more [likely] they are to develop whatever concession is provided to them.” (TSO-02) “What could be done with palm oil stranded assets? I think the answer is not much, if the land is already allocated under perkebunan, under an estate crop designation, [because] then you would need to change the underlying designation of the land. […] So you would need to change the underlying status of the land now, it is theoretically possible. But I think it’s probably highly unlikely.” (CON-04)
Compensating for past deforestation liabilities 14 interviews, 30 references	Paradox: As deforestation cut-off dates increasingly lie in the past, companies that engaged in clearing incur past liabilities which need to be addressed before re-entering supply chains. Proposed solutions such as the RSPO RaCP are perceived as cost-prohibitive, as growers may lack liquidity to pay for damages. Yet, they will be permanently locked out of major markets otherwise.	Environmental sustainability	Economic sustainability	“The people that should be financing [restoration] are the suppliers, because they are the ones with the liability. However, these suppliers, they don’t have the cash flow. All their collateral is already leveraged for loans. And there are far too many opportunities to actually just go sell to somebody else.” (CON-04)
Conserving nature on corporate plantations 14 interviews, 27 references	Paradox: Companies complying with ZDCs are tasked to preserve remaining forest and peatland within their concession. Beside the high monitoring and opportunity cost, this may also contradict the Indonesian Abandoned Land policy, which tasks companies to convert all allocated area to productive uses under the threat of permit revocation. If such permits are revoked, it is likely that the entire area will be converted, negating the aim of deforestation prevention.	Environmental sustainability	Economic sustainability *[reinforced by public policy]*	“Look, we’ve got massive areas of conservation, it’s almost 66,000 hectares […]. If you don’t allow us to develop a limited area, that area will be taken back by the government. And that will be demolished in no time. And it will be logged out and all the timber will be gone. So your true moral understanding of conservation or no deforestation doesn’t mean anything [in that case].” (GRO-02) “That’s been the huge spanner in the works all along. Especially in Indonesia, where the government says this is the land we’re giving you. Yeah, it’s got forest on it, we expect you to clear it, and develop it. Historically, before the moratorium, if they were to comply with RSPO, they would then not be fulfilling their agreement with the government and the government would then take back that land and give it to someone else who would develop it.” (NGO-07)
Verifying grievances 21 interviews, 34 references	Tension: Before grievances are addressed, their veracity needs to be verified. For cost reasons, this is often based on self-reported data by the offending company, or delegated to intermediaries. Yet both types of actors have conflicting interests to protect their business; thus alternative evidence may be presented, delaying or preventing ZDC enforcement. Such delays are likely to dissatisfy NGO stakeholders, which will continue to release reports that damage companies’ reputations.	Vertical cooperation	Competition	“We do have to check the validity of it. Which might mean getting some third party evidence, or, well it does mean speaking directly to them. […] Even if it’s a deforestation case, it’s often more complicated, because it’s not them, it’s outside their boundaries, it’s the community, it’s a financial [actor], there’s always a process to go through to understand sort of causality and kind of ultimately culpability.” (TRA-03) “I know that there are problems with some traders because the staff members, they are just quite lenient with the growers. So you’ll often get feedback saying, Oh, we spoke to this grower and they did an assessment and it is not palm. Why are you accepting that? If anything, you need to show proof, whatever assessment done needs to be peer reviewed and publicly available.” (NGO-08)
Choosing the right grievance response 31 interviews, 74 references	Paradox: Once grievances are verified, offending suppliers are usually asked to stop clearing and engage in remediation actions. If engagement is unsuccessful, ZDC companies may exclude the supplier. Such a step—crucial for accountability—may disrupt supply, increase prices, and reduce traded volumes, which has a negative commercial impact especially on midstream actors. The alternative, however, is to continue allowing deforestation in the supply chain (and risk market repercussions).	Environmental sustainability	Economic sustainability	“It’s a big thing to suspend one of your suppliers because that volume is essentially lost for the next couple of years. There is no volume on the markets, which you could easily take [up] to fill that void.” (LISC-05) “Commercial and sustainability, I think they don’t really go hand in hand. But we are trying to make them go hand in hand somehow.” (TRA-04) “[Not dropping suppliers] is also about not destroying themselves, keeping their profitability, and effectively desiring a level playing field.” (CON-04)
Coordinating responses to non-compliance 17 interviews, 44 references	Paradox: To increase the effectiveness of ZDC market pressure, buyers with ZDCs should set the same rules on market exclusion and conditions for re-entry. But anti-trust law prohibits them from jointly targeting specific suppliers. Furthermore, delaying a response compared to one’s competitor allows the company to gain a competitive edge in maintaining its sourcing base.	Horizontal cooperation	Competition *[reinforced by public policy]*	“The successes that you see, it’s when there’s been collective action by all buyers. The problem is that there’s issues of anti-competition and anti-trust, so the Indonesian Palm Oil pledge, that fell apart and that scared a lot of people. Buyers will often not want to announce that they have stopped buying from a grower because of the political ramifications.” (NGO-08) “As far as selectively going against a supplier or putting collective pressure, we’re not allowed to do that.” (CGM-05)

**Table 10:** Select paradoxes and an overview of approaches to manage such paradoxical tensions

Select paradoxical issues (see full descriptions in Table 9)	Creative approaches	Prioritization approaches	Defensive approaches
*Environmental-social paradoxes*			
Preventing deforestation outside of concessions	Mapping, local supply chain partnerships, legalization, social forestry	Prioritize own over third-party supply De-prioritize focusing on land barons	Separate smallholder (capacity building) issue from deforestation issue Advocate for ‘smallholder’ loopholes that cover others
*Environmental-economic paradoxes*			
Sharing supply base maps	Build trust, deepened engagement with select suppliers	Focus on traceability to mill, postpone traceability to plantation	Offer alternative accountability than map sharing Lobby for transparency restrictions
Choosing the right grievance response	Top-down leadership, KPI alignment	Prioritize traceability over engagement, and engagement over exclusion	Question idea of group-level accountability, discount exclusion benefits Use internal conflicts to justify inaction in response to deforestation events
Monetizing stranded assets and legacy cases	Scope out opportunities for land swaps Explore carbon markets as alternative revenue stream	Postpone development until prices rise; lobby for exceptions in the meantime	Advocate for exceptions for legacy cases Negotiate case-by-case Use shadow companies Link to development issue through advocating for ‘high forest cover landscape’ exceptions Exploit legal mandate to clear
*Cooperation-competition paradoxes*			
Coordinating responses to non-compliance	Alignment without coordination (follow the leader) Informal industry groups	Cooperate on less contentious issues (e.g., reporting norms) rather than information sharing	Reference to competition law to justify non-alignment or non-participation in collective action Lobby for government intervention with respect to competition law

**Table 11:** Leading refiners and traders of palm oil and their achievements in palm oil traceability, monitoring, and grievance resolution in 2020

Company name	SEA palm oil refining capacity (million MT/year; % of total)	Volume sourced in 2020 (million MT; % of world trade)	% of all palm oil supply RSPO-certified	% of supply traceable to mill level (2020)	% of supply from own mills traceable to plantation level (2020)	% of supply from third-party mills traceable to plantation level (2020)	Deforestation monitoring in supplier operations	Grievance procedure available	% of grievances solved (total number of grievances)
*Top 10 refiners*									
Wilmar	18.9 (24%)	25.3 (50%)	7%	98%	100%	14%	Yes (EQ, GFW)	Yes	21% (57)
Musim Mas	7.5 (10%)	10.4 (20%)	11%	100%	100%	80%	Yes (EQ)	Yes	42% (33)
Golden Agri Resources	6.1 (8%)	8.2 (16%)	16%	100%	100%	90%	Yes (SAT)	Yes	69% (32)
Apical Group	4.2 (5%)	8.9 (18%)	3%	100%	–	99%	Yes (GFW)	Yes	38% (16)
Mewah International	3.2 (4%)	2.5 (5%)	9%	100%	100%	80%	Yes (GFW)	Yes	50% (14)
FGV Holdings (Felda)	2.5 (3%)	3.8 (7%)	14%	100%	82%		Yes (GFW)	Yes	N/A
Sime Darby	2.5 (3%)	2.9 (6%)	64%	97%	50%		Internal	Yes	25% (53)
HSA Group/Pacific Inter-Link	2.4 (3%)		2.4%	100%	–	0%	No	Yes	22% (18)
IOI Group	2.4 (3%)	2.4 (5%)	28%	100%	100%	21%	Yes (EQ)	Yes	47% (30)
Permata Hijau Group	2.2 (3%)	2.6 (5%)	5%	100%	0%	0%	No	Yes	N/A
*Other major traders*									
Cargill		3.3 (6%)	22%	99%	48% of global supply		Yes (GFW)	Yes	30% (76)
Bunge		1.9 (4%)	35%	97%	–	77%	Yes (EQ, SAT)	Yes	44% (61)
Louis Dreyfus	1.2 (2%)	1.8 (4%)	5%	98%	–	78%	Yes (EQ, SAT, GFW)	Yes	39% (61)
Archer Daniels Midland Co		1.7 (3%)	11%	99%	–	45%	Via suppliers	Yes	37% (90)

Palm oil refining capacity in Indonesia/Malaysia from ten Kate et al. (2020). Volumes sourced (in metric tonnes, MT) represent all palm oil and palm oil products, including crude palm oil, crude palm kernel oil, derivatives refined from CPO and CPKO, and crude palm kernel expeller. From RSPO ACOP (RSPO, 2022). Palm world trade volumes approximated via global aggregate imports (palm oil and palm kernel oil), in MT, from FAO Stats (FAO, 2022). Given extensive inter-company trade between large companies, percentage values should not be read as mutually exclusive (and thus not summed to arrive at market coverage). Monitoring acronyms: GFW = Global Forest Watch Pro, SAT = Satelligence, EQ = Earthqualizer. Percentage of grievances solved from Mighty Earth’s Rapid Response Monitoring System; only includes grievances raised by Mighty Earth (Mighty Earth, 2019). All other indicators from November 2021 SPOTT scores (SPOTT, 2022)

## Footnotes

1 In the following, we will use ZDC (zero-deforestation commitment) and NDPE (no deforestation, no peat, no exploitation) policy implementation interchangeably, as this reflects the language used by practitioners. It should be noted that the scope of NDPE policies goes beyond only preventing deforestation and also targets peatland development and human rights abuses, although—as the study will show—most implementation efforts have focused on land use change, while the exploitation dimension has been relatively neglected.

2 Legally, smallholders are defined in Indonesia as individuals holding less than 25 hectares, though most households only manage 1–2 hectares (Daemeter Consulting 2015b; ISEAL Alliance 2019).
